# Bacterial Tyrosinase Inhibition, Hemolytic and Thrombolytic Screening, and In Silico Modeling of Rationally Designed Tosyl Piperazine-Engrafted Dithiocarbamate Derivatives

**DOI:** 10.3390/biomedicines11102739

**Published:** 2023-10-10

**Authors:** Ameer Fawad Zahoor, Freeha Hafeez, Asim Mansha, Shagufta Kamal, Muhammad Naveed Anjum, Zohaib Raza, Samreen Gul Khan, Jamila Javid, Ali Irfan, Mashooq Ahmad Bhat

**Affiliations:** 1 Department of Chemistry, Government College University Faisalabad, Faisalabad 38000, Pakistan; fawad.zahoor@gcuf.edu.pk (A.F.Z.); freeha.hafeez@riphahfsd.edu.pk (F.H.); samreengul@gcuf.edu.pk (S.G.K.); raialiirfan@gmail.com (A.I.); 2 Department of Chemistry, Riphah International University Faisalabad, Faisalabad 38000, Pakistan; 3 Department of Biochemistry, Government College University Faisalabad, Faisalabad 38000, Pakistan; shaguftakamal81@gmail.com; 4 Department of Applied Chemistry, Government College University Faisalabad, Faisalabad 38000, Pakistan; anjumccj@hotmail.com; 5 Department of Chemistry, School of Physical Sciences, University of Adelaide, Adelaide, SA 5000, Australia; zohaib.raza@adelaide.edu.au; 6 Department of Chemistry, University of Sialkot, Sialkot 51310, Pakistan; jamila.javaid@uskt.edu.pk; 7 Department of Pharmaceutical Chemistry, College of Pharmacy, King Saud University, Riyadh 11451, Saudi Arabia

**Keywords:** Tosyl piperazine, dithiocarbamate derivatives, ultrasonic-assisted green synthesis, bacterial tyrosinase inhibition, hemolytic activity, thrombolytic, molecular docking

## Abstract

Piperazine is a privileged moiety that is a structural part of many clinical drugs. Piperazine-based scaffolds have attracted the attention of pharmaceutical and medicinal scientists to develop novel, efficient therapeutic agents owing to their significant and promising biological profile. In the current study, an ecofriendly ultrasonic-assisted synthetic approach was applied to achieve a novel series of 1-tosyl piperazine dithiocarbamate acetamide hybrids **4a**–**4j,** which was evaluated for in vitro tyrosinase inhibition and thrombolytic and hemolytic cytotoxic activities. Among all the piperazine-based dithiocarbamate acetamide target molecules **4a**–**4j**, the structural analogs **4d** displayed excellent tyrosinase inhibition efficacy (IC_50_ = 6.88 ± 0.11 µM) which was better than the reference standard drugs kojic acid (30.34 ± 0.75 µM) and ascorbic acid (11.5 ± 1.00 µM), respectively, which was further confirmed by in silico induced-fit docking (IFD) simulation Good tyrosinase activities were exhibited by **4g** (IC_50_ = 7.24 ± 0.15 µM), **4b** (IC_50_ = 8.01 ± 0.11 µM) and **4c** (IC_50_ = 8.1 ± 0.30 µM) dithiocarbamate acetamides, which were also better tyrosinase inhibitors than the reference drugs but were less active than the **4d** structural hybrid. All the derivatives are less toxic, having values in the 0.29 ± 0.01% to 15.6 ± 0.5% range. The scaffold **4b** demonstrated better hemolytic potential (0.29 ± 0.01%), while a remarkably high thrombolytic chemotherapeutic potential was displayed by analog **4e** (67.3 ± 0.2%).

## 1. Introduction

**Figure 1:**
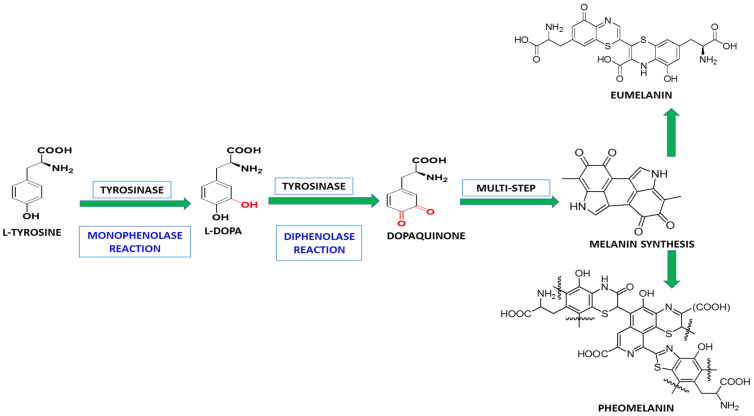
Biosynthetic route of melanin synthesis.

Tyrosinases (TYRs) and catechol oxidases comprise binuclear copper enzymes called polyphenol oxidases (PPOs) [1]. Tyrosinase (E.C. 1.14.18.1) is a ubiquitous, naturally occurring, multi-copper-containing enzyme involved in the synthesis of various types of melanin pigments. This process is called melanogenesis and can be found in microorganisms (bacteria and fungi), mammals, and plants (fruits and vegetables) [2,3,4]. In the biosynthetic route of melanin synthesis, monophenol (L-tyrosine) can be converted into diphenol (L-DOPA) by utilizing tyrosinase as a catalyst (cresolase or monophenolase activity), and tyrosinase further catalyzes this intermediate product, L-DOPA, to convert it into dopaquinone (catecholase or diphenolase activity). The dopaquinone is converted into the final product melanin (brown, yellow, and black) after consecutive reactions in the multistep biosynthetic pathway, as described in Figure 1 [2,4,5].

**Figure 2:**
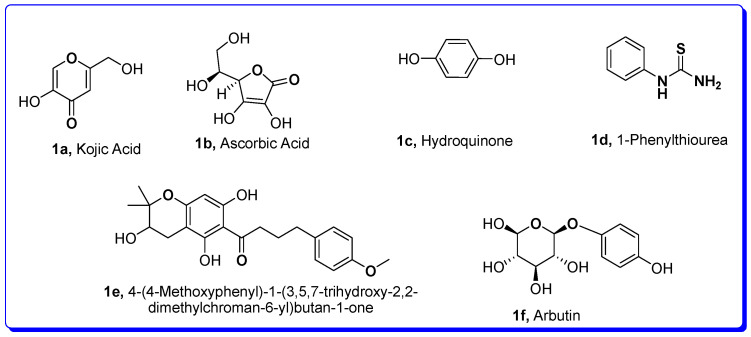
Structures of tyrosinase inhibitors.

Tyrosinase is of great interest as a potential anti-browning agent for fruits and vegetables [6], as a molting procedure for arthropods [7], and as a potent drug for the treatment of melanin hyperpigmentation [8]. Hence, tyrosinase inhibitors play a vital role in the fields of cosmetics, pharmaceuticals, and agriculture [9]. The structures of a few tyrosinase inhibitors are given in Figure 2.

**Figure 3:**
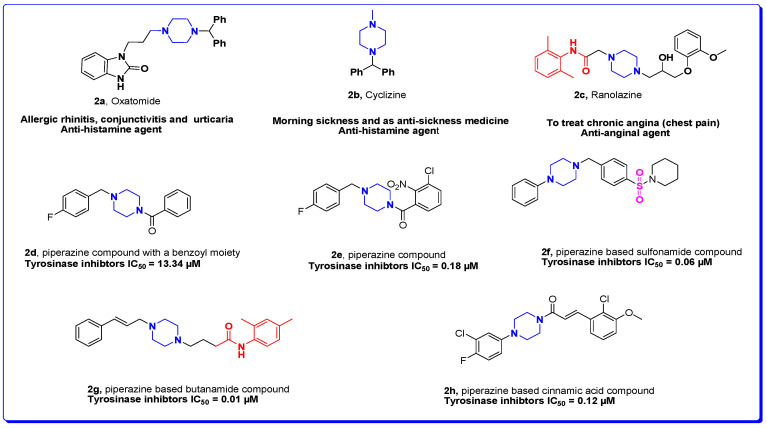
Structures of FDA-approved piperazine-based drugs.

Heterocycles such as oxadiazoles [4,10,11], quinoxalines [12], triazoles [10,13], pyrazoles [14], quinoxaline-sulfonamides [15], lamivudine [16], thiadiazoles [17,18], furans [4,10,11], and ciprofloxacin oxadiazole [19], etc., display a wide array of biological activities against different diseases, especially tyrosinase inhibitory activities [4,20]. Piperazine-based heterocyclic molecules [21,22,23] demonstrate a broad spectrum of biological and pharmacological chemotherapeutic potential [24], for instance, antimicrobial [25,26], antifungal [27], insecticidal [28], anti-oxidant [29], and anti-inflammatory [30,31], and they are currently also employed as anti-cancer agents [32]. The structures of some of the FDA-approved piperazine-based drugs are given in Figure 3.

Similarly, dithiocarbamates possess exceptional characteristics in the field of agrochemicals, such as their ability to act as herbicides, fungicides, insecticides, and pesticides [33]. Various organopharmacophores developed by inserting dithiocarbamates with organic molecules have been revealed as potent drugs for the treatment of various ailments. These dithiocarbamate hybrids exhibit anticancer [34], anti-tubercular, antifungal, antibacterial, antidiabetic, anti-obesity, anticonvulsant, antihelminth, and anti-Alzheimer activities [35,36].

**Figure 4:**
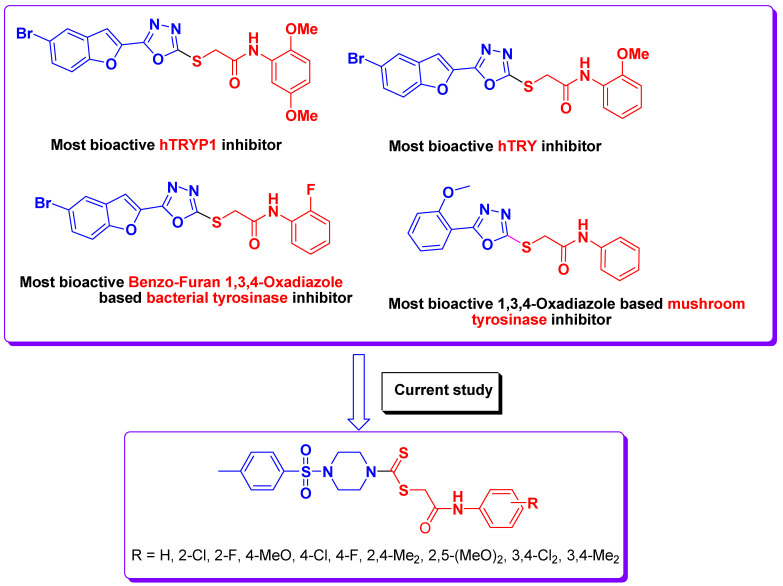
Rationale design on the basis of bioactive tyrosinase inhibitors.

Keeping in mind the various significant factors attributed to tyrosinase, the design and synthesis of potential tyrosinase inhibitors can be beneficial in understanding different life processes. Our research group already reported furan-tethered *N*-phenyl acetamide derivatives as tyrosinase inhibitors [4,20], and now in this current research work, the furan core is replaced with piperazine-dithiocarbamate, as depicted in Figure 4. Therefore, in this study, a library of 1-tosyl-substituted piperazine-based tyrosinase inhibitors was designed and synthesized. These derivatives were also examined for their inhibitory potential against tyrosinase and for their cytotoxic profile via hemolysis and thrombolysis.

## 2. Materials and Methods

### 2.1. Materials

The reagents and chemicals for the targeted scheme were obtained from Merck (Burlington, MA, USA), Fischer (Waltham, MA, USA) and Acros Organics (Guglielmo Marconi, Verona, Veneto, Italy) and were used as supplied. All solvents were of analytical grade and were purified by distillation before use in the experimental protocols. FT-IR (Fourier transform infrared spectra) were estimated on the Bruker OPUS FT-IR spectrometer by attenuated total reflection (Diamond ATR) on solid films. Meanwhile, ^1^H NMR and ^13^C NMR were recorded via the Bruker DPX-400 and at 500 MHz, AV400, or AV(III)400 (Bruker, Zurich, Switzerland) machines using deuterated chloroform (CDCl_3_) and were employed to report chemical shifts in ppm. The ESI-HRMS data were recorded using the Bruker Micro TOF-ESI (Bruker Daltonics, Germany) positive targeted mode. The elemental analysis (CHN) was performed on the CE-440 Elemental Analyzer (Exeter Analytical (Coventry, UK) Ltd.). Melting points were estimated on the Gallenkamp instrument (Fisons, Uckfield, UK). 

### 2.2. General Synthetic Procedure for Compounds

1-tosyl piperazine (1.0 eq.) was dissolved in the solution of NaOAc_aq._ (1.0 eq.) and CS_2_ (1.0 eq.) in 0.81 mL of methanol. To this mixture, a solution of the corresponding 2-bromo *N*-phenyl acetamide **3a**–**j** (Supplementary Figures S1–S20) (1.0 eq.) in 0.23 mL of methanol was added, which was then sonicated at 70 °C for 30 min. Precipitates were obtained after the completion of the reaction, which were filtered off, washed with distilled water, and purified by column chromatography (Ethyl Acetate/n-Hexane).

**2-Oxo-2-(phenylamino)ethyl 4-tosylpiperazine-1-carbodithioate (4a).**

Yield (75%), m.p. 200 °C; FTIR 3233 (NH), 1640 (C=O), 1521 (C=C), 1453 (CH_2_), 1234 (C=S), 1205 (S=O); ^1^HNMR (CDCl_3_ 400 MHz, chemical shift *δ*/ppm); 8.79 (s, 1-H, amine), 07.66 (doublet, 2-H, *J* = 8.0 Hz, Aryl-Hydrogen), 7.49 (doublet, 2-H, *J_H_* = 8.0 Hz, Aryl-Hydrogen), 07.37 (doublet, 2-H, *J_H_* = 4.0 Hz, Aryl-Hydrogen), 7.32 (doublet, 2-H, *J_H_* = 8.0 Hz, Aryl-Hydrogen), 7.11 (dd, 1-H, *J_H_* =4.0 Hz, Ar-H), 4.8 (bs, 2-H, piperazine), 4.2 (s, 2H, S-CH_2_), 4.1 (bs, 2H, piperazine), 3.1 (triplet, 4-H, *J_H_* =4.0 Hz, piperazine-H), 2.4 (s, 3-H, Me); ^13^CNMR (CDCl_3_ 100 MHz, chemical shift *δ*/ppm); 196.9 (CS), 166.5 (CO), 144.9, 138.0, 132.0, 130.0, 129.0, 127.7, 124.1, 120.1 (Ar-C), 46.2 and 45.5 (N-CH_2_ piperazine), 40.6 (S-CH_2_), 21.4 (CH_3_). HRMS calcd. 449.0902 found 449.0946 [M^+^] (100%). Anal Calcd. for C_20_H_23_N_3_O_3_S_3_: C = 53.43; H = 5.16; N = 9.35; Found C = 53.44; H = 5.17; N = 9.37.

**2-((2-Chlorophenyl)amino)-2-oxoethyl 4-tosylpiperazine-1-carbodithioate (4b).**

Yield = 80%, m.p. 180 °C; FTIR 3235 (NH), 1600 (C=O), 1520 (C=C), 1453 (CH_2_), 1234 (C=S), 1212 (S=O); ^1^HNMR (CDCl_3_ 400 MHz, chemical shift δ/ppm); 8.79 (s, 1-H, Amine), 8.30–8.36 (dd, 1-H, *J*_1_ = 8.0 Hz, *J*_2_ = 16.0 Hz Aryl-Hydrogen), 7.65 (doublet, 2-H, *J_H_* = 8.0 Hz, Aryl-Hydrogen), 7.62 (doublet, 1-H, *J_H_* = 8.0 Hz, Aryl-Hydrogen), 7.36 (doublet, 2-H, *J_H_* = 8.0 Hz, Aryl-Hydrogen), 7.32 (doublet, 1-H, *J_H_* = 8.0 Hz, Aryl-Hydrogen), 7.04–7.08 (dd, 1-H, *J*_1_ = 8.0 Hz, *J*_2_ = 8.0 Hz, Aryl-Hydrogen), 4.4 (bs, 2-H, piperazinyl-H), 4.2 (s, 2H, SCH_2_), 4.1 (bs, 2-H, piperazinyl H), 3.1 (triplet, 4-H, *J_H_* = 4.0 Hz, piperazinyl-H), 2.4 (s, 3-H, Me); ^13^CNMR (CDCl_3_ 100 MHz, chemical shift δ/ppm); 195.7 (CS), 166.6 (CO), 144.3, 134.8, 132.9, 132.2, 130.2, 129.2, 127.8, 124.9, 123.3, 121.8 (Ar-C), 45.9 and 45.5 (N-CH_2_ piperazine), 40.9 (S-CH_2_), 21.5 (CH_3_). HRMS calcd. 483.0572 found 483.0556 [M^+^] (100%). Anal Calcd. for C_20_H_22_ClN_3_O_3_S_3_: C, 49.63; H, 4.58; N, 8.68; Found C, 49.66; H, 4.59; N, 8.69.

**2-((2-Fluorophenyl)amino)-2-oxoethyl 4-tosylpiperazine-1-carbodithioate (4c).**

Yield 82%, m.p. 175 °C; IR 3235 (NH), 1655 (C=O), 1520 (C=C), 1453 (CH_2_), 1234 (C=S), 1212 (S=O); ^1^HNMR (CDCl_3_ 400 MHz, chemical shift *δ*/ppm); 8.79 (s, 1-H, Amine), 8.26 (dd, 1-H, *J*_1_ = 8.0 Hz, *J*_2_ = 16.0 Hz, Aryl-Hydrogen), 7.64 (doublet, 2-H, *J_H_* = 8.0 Hz, Aryl-Hydrogen), 7.37 (doublet, 2-H, *J_H_* = 8.0 Hz, Aryl-Hydrogen), 7.06 (m, 3-H, Aryl-Hydrogen), 4.48 (bs, 2-H, piperazinyl-H), 4.25 (s, 2-H, SCH_2_), 4.15 (bs, 2-H, piperazinyl-H), 3.37 (triplet, 4-H, *J* = 4.0 Hz, piperazinyl-H), 2.45 (s, 3-H, Me); ^13^CNMR (CDCl_3_ 100 MHz, chemical shift *δ*/ppm); 196.4 (CS), 166.3 (CO), 153.9, 151.6, 144.2, 138.5, 131.8, 129.8, 127.8, 126.2, 124.5, 121.5, 115.1 (Ar-C), 49.3 and 45.6 (N-CH_2_ piperazine), 40.5 (S-CH_2_), 21.5 (CH_3_). HRMS calcd. 467.0807 found 467.0851 [M^+^] (100%). Anal Calcd. for C_20_H_22_FN_3_O_3_S_3_: C, 51.37; H, 4.74; N, 8.99; Found C, 51.40; H, 4.77; N, 8.95.

**2-((4-Methoxyphenyl)amino)-2-oxoethyl 4-tosylpiperazine-1-carbodithioate (4d).**

Yield = 79%, m.p. 175 °C; FTIR 3233 (NH), 1645 (C=O), 1525 (C=C), 1452 (CH_2_), 1232 (C=S), 1220 (S=O); ^1^HNMR (CDCl_3_ 400 MHz, chemical shift *δ*/ppm); 8.64 (s, 1-H, Amine), 7.66 (doublet, 1-H, *J_H_* = 8.0 Hz, Aryl-Hydrogen), 7.61 (d, 1-H, *J_H_* = 8.0 Hz, Aryl-Hydrogen), 7.40 (doublet, 2-H, *J_H_* = 8.0 Hz, Aryl-Hydrogen), 7.26 (doublet, 2-H, *J_H_* = 8.0 Hz, Aryl-Hydrogen), 6.88 (doublet, 2-H, *J_H_* = 8.0 Hz, Aryl-Hydrogen), 4.47 (bs, 2-H, piperazinyl-H), 4.19 (s, 2-H, SCH_2_), 4.08 (bs, 2-H, piperazinyl-H), 3.80 (s, 3-H, OMe), 3.61 (t, 4H, *J_H_* = 4.0 Hz, piperazine), 2.47 (s, 3-H, Me); ^13^CNMR (CDCl_3_ 100 MHz, chemical shift *δ*/ppm); 196.9 (CS), 166.4 (CO), 156.8, 144.6, 132.3, 130.9, 130.0, 127.6, 121.4, 114.0 (Ar-C), 55.4 (OCH_3_), 46.9 and 45.6 (N-CH_2_ piperazine), 40.0 (S-CH_2_), 21.7 (CH_3_). HRMS calcd. 479.1007 found 479.1051 [M^+^] (100%). Anal Calcd. for C_21_H_25_N_3_O_4_S_3_: C, 52.59; H, 5.25; N, 8.76; Found C, 52.61; H, 5.27; N, 8.76.

**2-((4-Chlorophenyl)amino)-2-oxoethyl 4-tosylpiperazine-1-carbodithioate (4e).**

Yield 77%, m.p. 185 °C; FTIR 3235 (NH), 1633 (C=O), 1520 (C=C), 1453 (CH_2_), 1234 (C=S), 1210 (S=O); ^1^HNMR (CDCl_3_ 400 MHz, chemical shift *δ*/ppm); 8.89 (s, 1-H, amine), 7.66 (doublet, 2H, *J_H_* = 8.0 Hz, Aryl-Hydrogen), 7.63 (doublet, 2-H, *J_H_* = 4.0 Hz, Aryl-Hydrogen), 7.37 (doublet, 2-H, *J_H_* = 8.0 Hz, Aryl-Hydrogen), 7.23 (doublet, 2-H, *J_H_* = 4.0 Hz, Aryl-Hydrogen), 4.48 (bs, 2H, piperazine), 4.18 (s, 2H, S-CH_2_), 4.08 (bs, 2H, piperazine), 3.17 (t, 4H, *J* = 4.0 Hz, piperazine), 2.45 (s, 3-H, Me); ^13^CNMR (CDCl_3_ 100 MHz, chemical shift *δ*/ppm); 197.2 (CS), 166.8 (CO), 144.6, 136.6, 133.0, 132.0, 130.0, 129.0, 127.6, 121.0 (Ar-C), 50.5 and 45.2 (N-CH_2_ piperazine), 40.3 (S-CH_2_), 21.7 (CH_3_). HRMS calcd. 483.0572 found 483.0556 [M^+^] (100%). Anal Calcd. for C_20_H_22_ClN_3_O_3_S_3_: C, 49.63; H, 4.58; N, 8.68; Found C, 49.68; H, 4.61; N, 8.70.

**2-((4-Fluorophenyl)amino)-2-oxoethyl 4-tosylpiperazine-1-carbodithioate (4f).**

Yield = 85%, m.p. 190 °C; FTIR 3235 (NH), 1647 (C=O), 1520 (C=C), 1453 (CH_2_), 1234 (C=S), 1208 (S=O); ^1^HNMR (CDCl_3_ 400 MHz, chemical shift *δ*/ppm); 8.81 (s, 1-H, Amine), 07.63 (doublet, 2-H, *J_H_* = 8.0 Hz, Aryl-Hydrogen), 7.46 (doublet, 2-H, *J_H_* = 8.0 Hz, Aryl-Hydrogen), 7.31 (doublet, 2-H, *J_H_* = 8.0 Hz, Aryl-Hydrogen), 6.99 (doublet, 2-H, *J_H_* = 8.0 Hz, Aryl-Hydrogen), 4.49 (bs, 2H, piperazine), 4.18 (s, 2H, S-CH_2_), 4.12 (bs, 2H, piperazine), 3.17 (triplet, 4-H, *J_H_* = 4.0 Hz, piperazinyl-H), 2.42 (s, 3-H, Me); ^13^CNMR (CDCl_3_ 100 MHz, chemical shift *δ*/ppm); 197.0 (C=S), 166.6 (C=O), 161.0, 144.9, 134.2, 132.2, 130.5, 127.5, 121.8, 115.8 (Ar-C), 46.6 and 45.6 (N-CH_2_ piperazine), 40.2 (S-CH_2_), 21.5 (CH_3_). HRMS calcd. 467.0807 found 467.0851 [M^+^] (100%). Anal Calcd. for C_20_H_22_FN_3_O_3_S_3_: C, 51.37; H, 4.74; N, 8.99; Found C, 51.35; H, 4.75; N, 8.99.

**2-((2,4-Dimethylphenyl)amino)-2-oxoethyl 4-tosylpiperazine-1-carbodithioate (4g).**

Yield = 89%, m.p. 185 °C; FTIR 3230 (NH), 1600 (C=O), 1524 (C=C), 1450 (CH_2_), 1232 (C=S), 1215 (S=O); ^1^HNMR (CDCl_3_ 400 MHz, chemical shift *δ*/ppm); 9.47 (s, 1-H, amine), 7.61 (doublet, 2-H, *J_H_* = 8.0 Hz, Aryl-Hydrogen), 7.46 (doublet, 2H, *J_H_* = 8.0 Hz, Aryl-Hydrogen), 7.16 (doublet, 1-H, *J_H_* = 8.0 Hz, Aryl-Hydrogen), 6.99 (s, 1-H, Aryl-Hydrogen), 6.93 (doublet, 1-H, *J_H_* = 8.0 Hz, Aryl-Hydrogen), 4.32 (bs, 2-H, piperazinyl-H) 4.20 (s, 2-H, SCH_2_), 4.09 (bs, 2-H, piperazinyl-H), 3.02 (triplet, 4-H, *J_H_* = 4.0 Hz, piperazinyl-H), 2.45 (s, 3-H, CH_3_), 2.28 (s, 3H, CH_3_), 2.09 (s, 3H, CH_3_); ^13^CNMR (CDCl_3_ 400 MHz, chemical shift *δ*/ppm); 196.8 (CS), 165.6 (CO), 144.5, 134.8, 133.9, 132.2, 131.5, 130.5, 128.5, 126.5, 125.8 (Ar-C), 47.0 and 46.3 (N-CH_2_ piperazine), 40.9 (S-CH_2_), 21.9, 21.2 and 18.2 (CH_3_). HRMS calcd. 477.1215 found 477.1259 [M^+^] (100%). Anal Calcd. for C_22_H_27_N_3_O_3_S_3_: C, 55.32; H, 5.70; N, 8.80; Found C, 55.34; H, 5.74; N, 8.83.

**2-((2,5-Dimethoxyphenyl)amino)-2-oxoethyl 4-tosylpiperazine-1-carbodithioate (4h).**

Yield = 90%, m.p. 190 °C; FTIR 3231 (NH), 1635 (C=O), 1523 (C=C), 1453 (CH_2_), 1230 (CS), 1222 (S=O); ^1^HNMR (CDCl_3_ 400 MHz, chemical shift *δ*/ppm); 8.85 (s, 1-H, Amine), 8.04 (doublet, 1-H, *J_H_* = 12.0 Hz, Aryl-Hydrogen), 7.63 (d, 2H, *J_H_* = 8.0 Hz, Aryl-Hydrogen), 7.37 (doublet, 2-H, *J_H_* = 8.0 Hz, Aryl-Hydrogen), 7.23 (s, 1-H, Aryl-Hydrogen), 6.79 (doublet, 1-H, *J_H_* = 8.0 Hz, Aryl-Hydrogen), 4.46 (bs, 2H, piperazine), 4.24 (s, 2-H, SCH_2_), 4.12 (bs, 2H, piperazine), 3.84 (s, 3-H, OMe), 3.79 (s, 3-H, OMe), 3.10 (t, 4H, *J_H_* = 4.0 Hz, piperazine), 2.47 (s, 3-H, CH_3_); ^13^CNMR (CDCl_3_ 400 MHz, chemical shift *δ*/ppm); 196.4 (C=S), 166.3 (C=O), 153.9, 144.6, 142.57, 132.2, 130.2, 128.5, 127.5, 111.5, 109.1, 106.5 (Ar-C), 57.0 (OCH_3_) 55.6 and 45.9 (N-CH_2_ piperazine), 41.6 (S-CH_2_), 21.2 (CH_3_). HRMS calcd. 509.1113 found 509.1157 [M^+^] (100%). Anal Calcd. for C_22_H_27_N_3_O_5_S_3_: C, 51.85; H, 5.34; N, 8.24; Found C, 51.86; H, 5.34; N, 8.26.

**2-((3,4-Dichlorophenyl)amino)-2-oxoethyl 4-tosylpiperazine-1-carbodithioate (4i).**

Yield 85%, off-white solid, m.p 214 °C; IR 3230 (NH), 1636 (C=O), 1524 (C=C), 1450 (CH_2_), 1232 (C=S), 1213 (S=O); ^1^HNMR (CDCl_3_ 400 MHz, chemical shift *δ*/ppm); 9.00 (s, 1-H, Amine), 7.74 (s, 1-H, Aryl-Hydrogen), 7.64 (doublet, 2-H, *J_H_* = 12.0 Hz, Aryl-Hydrogen), 7.61 (doublet, 1-H, *J_H_* = 8.0 Hz, Aryl-Hydrogen), 7.43 (d, 2H, *J_H_* = 4.0 Hz, Aryl-Hydrogen), 7.38 (s, 1-H, Aryl-Hydrogen), 4.52 (bs, 2-H, piperazinyl-Hydrogen), 4.20 (s, 2H, S-CH_2_), 4.12 (bs, 2H, piperazine-H), 3.17 (t, 4H, *J* = 4.0 Hz, piperazine-H), 2.47 (s, 3H, CH_3_); ^13^CNMR (CDCl_3_ 400 MHz, chemical shift *δ*/ppm); 196.7 (C=S), 166.6 (C=O), 144.5, 137.2, 132.5, 131.8, 130.9, 129.9, 127.8, 121.5, 119.2 (Ar-C), 50.3 and 45.2 (N-CH_2_ piperazine), 40.3 (S-CH_2_), 21.5 (CH_3_). HRMS calcd. 517.0122 found 517.0166 [M^+^] (100%). Anal Calcd. for C_20_H_21_Cl_2_N_3_O_3_S_3_: C, 46.33; H, 4.08; N, 8.10; Found C, 46.34; H, 4.08; N, 8.11.

**2-((3,4-Dimethylphenyl)amino)-2-oxoethyl 4-tosylpiperazine-1-carbodithioate (4j).**

Yield = 80%, m.p. 180 °C; FTIR 3230 (NH), 1640 (C=O), 1524 (C=C), 1450 (CH_2_), 1232 (C=S), 1210 (S=O); ^1^HNMR (CDCl_3_ 400 MHz, chemical shift *δ*/ppm); 8.62 (s, 1-H, Amine), 7.66 (doublet, 2-H, *J_H_* = 8.0 Hz, Aryl-Hydrogen), 7.40 (doublet, 2-H, *J_H_* = 8.0 Hz, Aryl-Hydrogen), 7.24 (s, 1-H, Aryl-hydrogen), 7.17 (doublet, 1-H, *J_H_* = 8.0 Hz, Aryl-Hydrogen), 4.49 (bs, 2-H, piperazinyl-hydrogen), 4.20 (s, 2-H, SCH_2_), 4.12 (bs, 2-H, piperazinyl-Hydrogen), 3.20 (t, 4H, *J* = 4.0 Hz, piperazine-H), 2.45 (s, 3H, CH_3_), 2.24 (s, 3H, CH_3_), 2.16 (s, 3H, CH_3_); ^13^CNMR (CDCl_3_ 400 MHz, chemical shift *δ*/ppm); 197.4 (CS), 166.3 (CO), 144.6, 137.2, 135.5, 133.2, 131.8, 130.2, 127.8, 121.1, 117.1 (Ar-C), 46.6 and 45.9 (N-CH_2_ piperazine), 40.6 (S-CH_2_), 21.3, 20.2 and 19.1 (CH_3_). HRMS calcd. 477.1215 found 477.1259 [M^+^] (100%). Anal Calcd. for C_22_H_27_N_3_O_3_S_3_: C, 55.32; H, 5.70; N, 8.80; Found C, 55.35; H, 5.71; N, 8.81.

### 2.3. Biological Screening of 1-Tosyl piperazine-dithiocarbamate Hybrid Derivatives ***4a**–**j***

#### 2.3.1. Tyrosinase Inhibition Evaluation of 1-Tosyl piperazine-dithiocarbamate Hybrid Derivatives **4a**–**j**

The inhibition of bacterial tyrosinase (*Bacillus subtilis* NA2) was investigated by spectrophotometric methodology [4,37]. IC_50_ values were obtained using a spectrophotometer assay. The protocol involved the addition of 2 mL of 1/2 mM _L_-Dopa in a 67.0 mM PBS (phosphate buffer saline) solution of pH 6.8 to the 800 µL solution of target molecules in DMSO. A total of 0.1 mL of the enzyme solution (tyrosinase) was dissolved in this mixture, and the starting rate of linear increase was measured in the optical density after 10, 20, and 30 min of incubation time, respectively. Thus, optical density increases or decreases with the presence of dopachrome. The inhibitory effect was shown by percentage inhibition, and the results were compared with the control.

#### 2.3.2. Hemolytic Activity of 1-Tosyl piperazine-dithiocarbamate Hybrid Derivatives **4a**–**j**

The hemolytic potential of this class of piperazine-based molecules was investigated according to the reported methodology [38]. A blood sample of 5 mL of volume from albino mice was taken and subjected to centrifugation at 1000 rpm for 5 min. RBC pellets were secluded and splashed 3–4 times using chilled buffer solutions that had pH values of 7.4, such as the phosphate buffer saline (PBS). A solution sample in a volume of 20 µL (10 mg/mL) was taken and added to a 180 µL RBC pellet. The sample tube was incubated for half an hour at 37 °C. A sample tube was taken from the incubator and lowered to room temperature in an ice bath for 5 min. The sample was again centrifuged at 13,000 rpm for 5 min. A total of 100 µL of supernatant was collected from each tube and diluted with the ice-cold phosphate buffered saline. DMSO and ABTS ((2,2′-azino-bis(3-ethylbenzothiazoline-6-sulfonic acid)) served as the negative and positive controls, respectively. Absorbance was recorded at 517 nm. The hemolytic effect was calculated in percentages. The following formula was employed:

$$\%age\;hemolysis=\frac{Absorbance\;of\;sample-Absorbance\;of\;negative\;control}{Absorbance\;of\;positive\;control}\times 100$$

#### 2.3.3. Thrombolytic Activity of 1-Tosyl piperazine-dithiocarbamate Hybrid Derivatives **4a**–**j**

The thrombolytic potential of these target derivatives was determined via the literature’s methodology [38]. Blood samples were collected from albino mice and relocated to numerous cleaned and weighed-up eppendorf tubes. We incubated these eppendorves at 37 °C for clot formation. Blood serum was cast-off from these tubes and weighed in the eppendorf tubes to calculate the preliminary weight of the clot (clot weight = weight of the eppendorf with the clot minus the weight of the empty eppendorf). The eppendorf tubes were filled with 100 µL of a solution of synthesized samples in DMSO and were incubated at 37 °C for 3 h. In this methodology, ABTS was used as a positive control, while water was used as a negative control. Serum was again removed and weighed in the eppendorf tubes to measure clot lysis. The results were demonstrated as percentages [23].

$$\%age\;thrombolysis=\frac{Initial\;clot\;weight-Final\;clot\;weight}{Initial\;clot\;weight}\times 100$$

#### 2.3.4. Molecular Docking Studies of 1-Tosyl piperazine-dithiocarbamate Hybrid Derivative **4d**

The compound **4d**, with the lowest inhibitory constant (Ki), was in silico modelled in induced-fit docking (Molecular Operating Environment (MOE) 2015) [39]. Crystallized structure of tyrosinase was accessed from Protein databank (PDB ID: 2Y9X), and served as receptor in simulation (https://www.rcsb.org accessed on 29 July 2023) [40]. Kojic acid (CID: 3840) was used as the standard in the IFD simulation, and its 3D conformer was obtained from the PubChem database. The macromolecule was prepared with a structural preparation module to fix its structural errors, and the Protonate 3D module was used to optimize its protonation state in order to withstand the molecular mechanic refinement of the docked poses. The molecular system was energy minimized with the Amber10:EHT forcefield to add tether restraints. The Site Finder module was used to specify the binding site in the vicinity of the co-crystalized ligand, and compounds were docked with the Triangular Matcher Placement method using the London dG scoring function to rank the docked poses. The docked poses were further refined with the induced-fit docking method and were ultimately ranked with the GBVI/WSA scoring function. The docking pose with the lowest binding energy (i.e., S) was selected to simulate the complexation and interactions of the ligands within the binding pocket of tyrosinase in Discovery studio visualizer v17.2. The apo conformation of the X-ray crystalized compound was used in cognate redocking to validate the docking protocol.

## 3. Results and Discussion

### 3.1. Synthetic Chemistry of 1-Tosyl piperazine-dithiocarbamate Hybrid Derivatives ***4a**–**j***

The synthesis of titled 1-tosyl piperazine-dithiocarbamate hybrid derivatives **4a**–**j** was achieved by employing the following methodology: The precursor 1-tosyl piperazine **2** was prepared by treating anhydrous piperazine **1** with *p*-toluenesulfonyl chloride in dichloromethane (DCM). Furthermore, different substituted *N*-phenyl bromoacetamides **3a**–**j** were obtained by reacting substituted aryl amines with bromoacetyl bromide, pyridine (Py), or DCM as the reaction medium. Finally, 1-tosyl piperazine **2** was coupled with various substituted *N*-phenyl bromoacetamides **3a**–**j** under ultrasonic conditions using carbon disulfide (CS_2_) and sodium acetate (NaOAc) to obtain the target hybrid derivatives **4a**–**j** (Supplementary Figures S1–S20) in a 75–90% yield [22] (Scheme 1). 

### 3.2. Spectral Characterization of the Representative and Most Bioactive Derivative ***4d***

The representative derivative **4d** was investigated for structure elucidation via FTIR, ^1^H NMR, ^13^C NMR, and MS. M^+^ at *m*/*z*: 479.1051. For the study of different functional moieties in FT-IR, various absorption bands were perceived at υ: 3233 (amino), 1645 (C=O), 1525 (C=C), 1452 (CH_2_), 1232 (CS), and 1220 (S=O). In the ^1^H NMR spectrum, the signals for N-H and methylene of amide were observed at 8.6 and 4.1 ppm, respectively. The most upfield signal was depicted by the methyl group at the aromatic ring as a singlet at 2.45, while the OMe functional group at the aromatic ring was reverberated at 3.7 ppm as a singlet. Furthermore, the H-2 and H-3 of the piperazine appeared as broad singlets at 4.0 and 4.4 ppm, respectively. Meanwhile, the H-4 and H-5 of the piperazine depicted their signal as a triplet at 3.1 ppm (J = 4.0 Hz). The aromatic protons H-1’ and H-2’ appeared at a chemical shift (δ) of 7.3 ppm with a coupling constant of J = 8.0 Hz as doublets. However, the aromatic protons Hydrogen-3′ and Hydrogen-4 were depicted as d at 7.63 ppm with a coupling constant of J = 8.0 Hz and a 7.61 ppm chemical shift at a coupling constant of J = 8.0 Hz, respectively. At a chemical shift of 7.4 ppm, along with a coupling constant of J = 8.0 Hz, two aryl hydrogens, Hydrogen-5′ and H-6, appeared as doublets, while at 6.8 ppm (J = 8.0 Hz), two hydrogens resonated as doublets.

**Figure 5:**
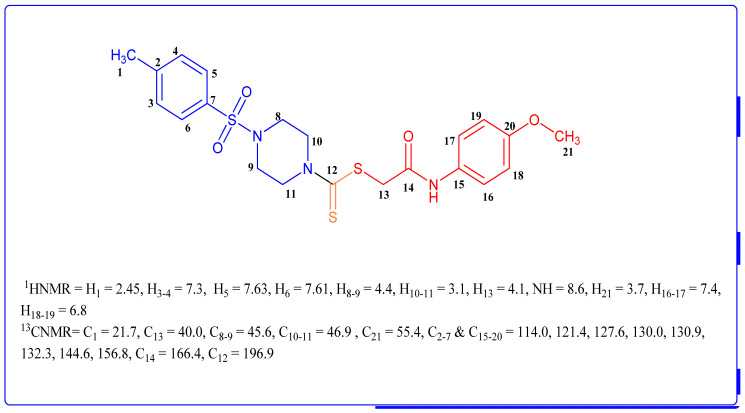
^1^HNMR and ^13^CNMR elucidation of representative molecule **4d** with chemical shifts (ppm).

The ^13^CNMR spectroscopy method was employed to inspect the carbon scaffold of **4d**. All 21 carbons displayed their signals in the spectrum. The most downfield signal was shown at 196.9 ppm, which belongs to C=S and confirmed the presence of dithiocarbamate. The presence of the acetamide motive was inveterately confirmed by the signals of the carbonyl and methylene groups, which appeared at 166.4 and 40.0 ppm, respectively. The Me group substituted at the aromatic ring showed up with the most upfield signal at 21.7 ppm. The 4-methoxy phenyl ring linked with acetamide depicted signals for four methine groups at 114.0 ppm for C-2′ and C-3′ and at 121.4 ppm for C-4′ and C-5, while for the substituted aryl ring, two signals appeared: at 130.9 for C-N and at 156.8 for C-OMe. The aryl ring attached to the sulfonic group depicted signals for substituted carbons at 144.6 ppm for C-S and at 132.3 ppm for C-Me. Furthermore, methine signals were resonated for C-8′ and C-9′ at 127.6 ppm and for C-10′ and C-11′ at 130.0 ppm (Figure 5). Other synthesized derivatives of this series were also structurally characterized by a similar methodology.

### 3.3. Biological Screening of 1-Tosyl piperazine-dithiocarbamate Hybrid Derivatives ***4a**–**j***

#### 3.3.1. Tyrosinase Inhibition Evaluation of 1-Tosyl piperazine-dithiocarbamate Hybrid Derivatives **4a**–**j**

**Figure 6:**
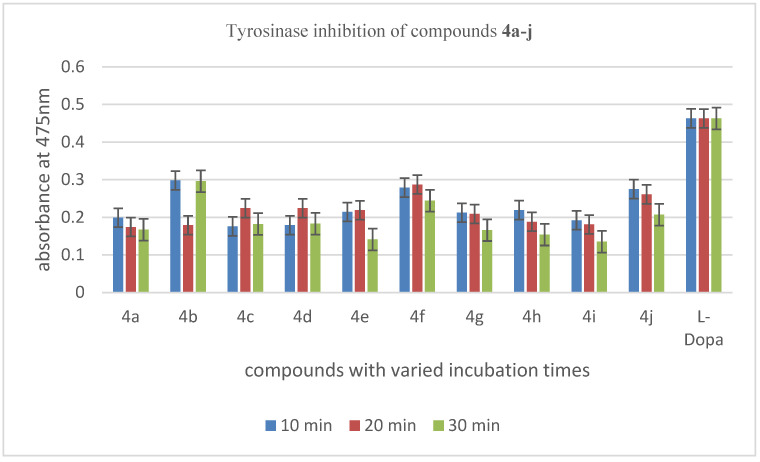
Tyrosinase inhibition with varied incubation times.

All the target derivatives were investigated for tyrosinase (*Bacillus subtilis* NA2) inhibition using an in vitro methodology [4,37]. The synthesized molecules exhibited a promising tyrosinase inhibition potential by means of IC_50_ values fluctuating between 6.88 and 34.8 µM and compared with the reference drug, kojic acid, with an IC_50_ of 30.34 ± 0.75 µM. When the IC_50_ values of the derivatives **4a**–**j** were compared, the results showed that the occurrence of an electron-withdrawing group (EWG) or electron-donating group (EDG) attached to the Ph-group, in general, enhanced the tyrosinase inhibition potential of the molecules. The position of the functional group also has an influence on the efficacy of the inhibition potential of target derivatives. Compound **4d** exhibited better inhibitory activity, showing an IC_50_ of 6.88 µM, and the methoxy substituent (electron donating through resonance/conjugation effect) attached to the *p*-position of the Ph-group is responsible for this effect (Table 1, entry 4). Meanwhile, derivative **4a**, without any attachment at the Ph-ring, showed the least efficacy among the target molecules with an IC_50_ of 34.8 µM (Table 1, entry 1). Other derivatives, such as **4c**, **4e,** and **4g**, also showed a better inhibition potential with IC_50_s of 8.1 µM, 11.11 µM, and 7.24 µM, respectively (Table 1, entries 3, 5, and 7). The inhibition potential of these derivatives was also studied at varied incubation times. Among the synthesized derivatives, the highest optical density was observed for **4b** after 30 min of incubation time, which can be attributed to the chloro group on the Ph-ring being responsible for this effect. Furthermore, the results reveal that the derivatives **4a**, **4g**, **4h**, **4i,** and **4j** showed a linear decrease in optical density as the incubation time increased from 10 to 30 min (Figure 6).

#### 3.3.2. Hemolytic Activity of 1-Tosyl piperazine-dithiocarbamate Hybrid Derivatives **4a**–**j**

**Figure 7:**
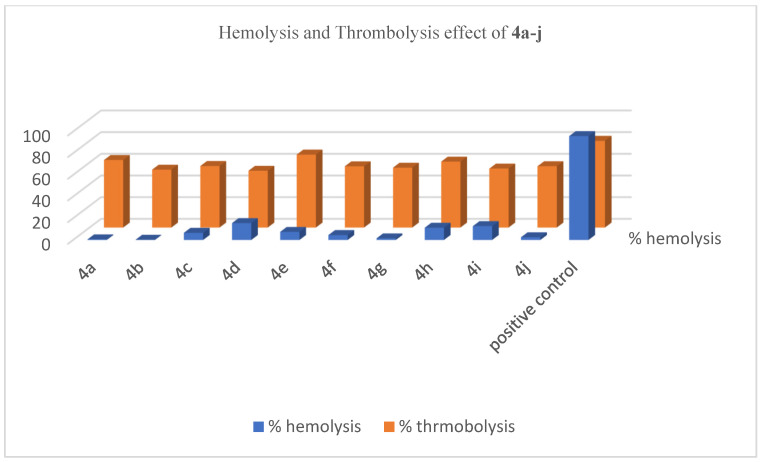
Graphical representation of hemolytic and thrombolytic effect of **4a**–**j**.

All the synthesized derivatives of this series 4a–j were evaluated for their cytotoxic potential via hemolysis using an in vitro technique [22,41]. The results from Table 1 demonstrate that 4b proved to be the least toxic compound, with 0.29% hemolysis, among all the target derivatives. The reason for this better activity is the EWG chloro moiety at the *o*-position of the phenyl ring. Among the other derivatives, compounds 4a (0.55%), 4g (1.53%), and 4j (2.56%) exhibited a moderate potential. Among the analogs, compounds 4d and 4i expressed the highest toxicity at 15.6% and 12.8%, respectively (Figure 7).

#### 3.3.3. Thrombolytic Activity of 1-Tosyl piperazine-dithiocarbamate Hybrid Derivatives **4a**–**j**

All the derivatives were also studied for in vitro thrombolytic assay [23,38]. However, on exploring the thrombolytic potential of these derivatives, the results indicate that all the derivatives exhibited a moderate potential. Among the synthesized derivatives, the highest potential was shown by the **4e** (67.3 ± 0.2) electron-withdrawing chloro functional group, which is responsible for this effect. Meanwhile, **4h** (60.83 ± 0.14) exhibited a moderate effect, and the reason that accounts for this effect is the 2,5-dimethoxy substituent. The results from Table 1 also show that **4d** showed the least potential (52.4 ± 0.2) (Figure 7).

#### 3.3.4. SAR (Structure Activity Relationship) Studies of 1-Tosyl piperazine-dithiocarbamate Derivatives **4a**–**j**

**Figure 8:**
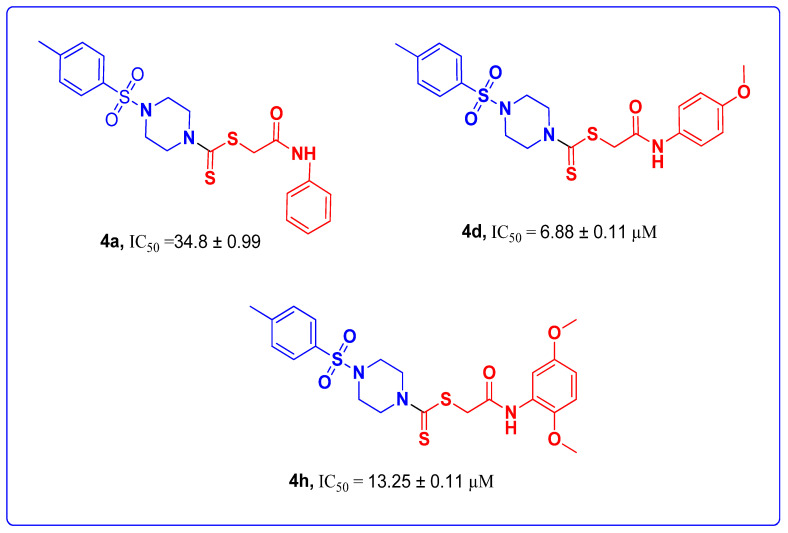
SAR studies of derivatives **4a**, **4d**, and **4h**.

The synthesized derivatives **4a**–**j** were investigated for SAR studies, depending on the nature of the substituents on the Ar-ring of the *N*-phenyl derivatives of acetamide, to gain information regarding the tyrosinase inhibition of these molecules. The inhibition potentials of the derivatives in this study reveal that generally, electron-donating substituents attached to an aryl ring enhance the effect. Among these synthesized derivatives, **4a**, with an unsubstituted phenyl ring, is the least active with an IC_50_ of 34.8 ± 0.99 µM. The attachment of the electron-donating and electron-withdrawing functional groups at different positions on the aryl ring has a remarkable effect on the tyrosinase inhibitory potential. Derivative **4d**, having an MeO group (electron donating through resonance/conjugation effect) at the *p*-position of the aromatic ring, showed an excellent inhibition value (IC_50_ = 6.88 ± 0.11 µM) among all the target derivatives. However, unexpectedly, a decrease in potential was observed when a second MeO group was installed at position 5 (2,5-dimethoxy phenyl) of the aromatic ring (IC_50_ = 13.25 ± 0.11 µM) (Figure 8).

**Figure 9:**
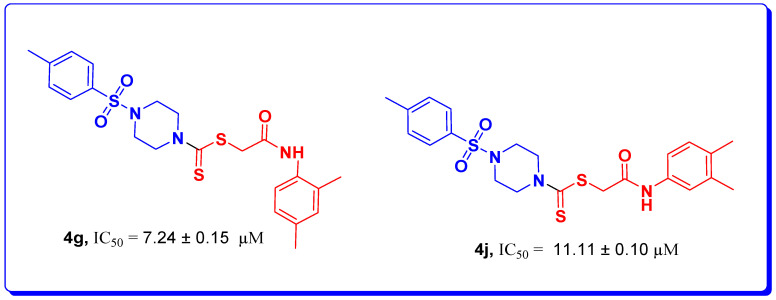
SAR studies of molecules **4g** and **4j**.

Similarly, the presence of an electron-donating methyl group on the phenyl ring also had a positive influence on the inhibitory potential. Derivative **4g**, having 2,4-dimethyl groups, was the second most active compound (IC_50_ = 7.24 ± 0.15 µM) among all the derivatives. A slight change in the position of the methyl functional group resulted in a decrease in inhibitory potential, as in, for example, the case of **4j** (IC_50_ = 11.11 ± 0.10 µM), where the position of the methyl group from *ortho*-*para* (as in **4g**) is switched to *meta*-*para* and a decrease in potential is observed. This suggests that e-donating groups at the *ortho*-*para* position have more interactions as compared to the *meta* position and account for a better inhibition potential (Figure 9).

**Figure 10:**
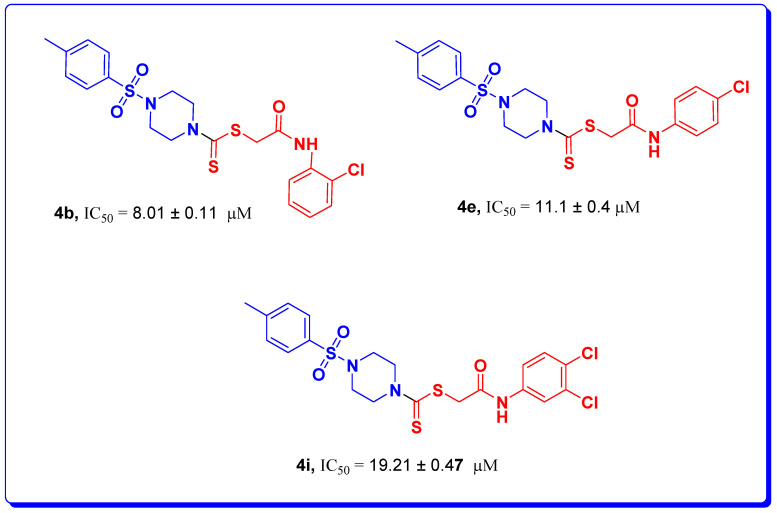
SAR studies of molecules **4b**, **4e**, and **4i**.

**Figure 11:**
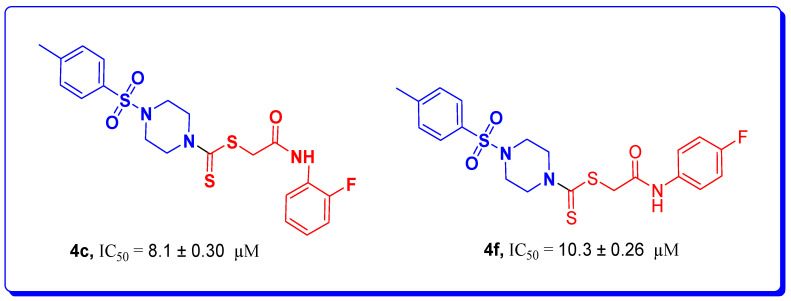
SAR studies of molecules **4c** and **4f**.

The inhibition potential of derivative **4b** (IC_50_ = 8.01 ± 0.99 µM), having Cl at the *o*-position, was considerable, while a reduction in potential was expressed when the chloro motif was switched at the *p*-position on the Ph-ring, as in case of **4e** (IC_50_ = 11.1 ± 0.4 µM). Furthermore, derivative **4i**, bearing two chloro substituents at the *meta*-*para* position, showed a decrease in inhibitory potential (IC_50_ = 19.21 ± 0.47 µM) (Figure 10). Derivative **4c**, with a flouro group at the *o*-position on the phenyl ring, expressed a considerable potential (IC_50_ = 8.1 µM), while the potential was slightly decreased in **4f** (IC_50_ = 10.3 µM), which has a flouro functional group at the *p*-position (Figure 11). It can be suggested that the electron-donating group on the Ph-ring tends to enhance the potential at the *p-position*.

#### 3.3.5. Molecular Docking Studies of 1-Tosyl piperazine-dithiocarbamate Derivative **4d**

**Figure 12:**
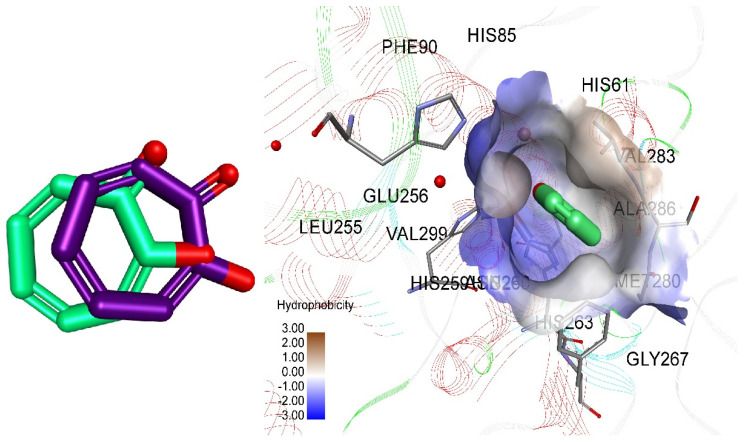
Validation of IFD protocol. Cognate-redocking of co-crystalized ligand (Green) aligned with its docked pose generated in IFD protocol (left): (Binding pocket of 2Y9X with hydrophobicity map (right).

The molecular docking screening of the most bioactive 1-tosyl piperazine-dithiocarbamate derivative **4d** was performed by using the induced-fit docking (IFD) protocol. The cognate redocking yielded a pose with a 1.6 Å root mean square deviation (RMSD) to its apo conformation and validated the docking protocol (Figure 12). 

The IFD simulation resulted in ligand complexation with a negative binding affinity and highlighted **4d** as a potential tyrosinase ligand with a superior binding affinity within its binding pocket. Kojic acid [42] was found to bind in the tyrosinase binding pocket with −4.84 ΔG (Kcal/mol) and provided the standard threshold for the IFD (Table 2). Interestingly, **4d** stabilized conformation with −7.3 ΔG (Kcal/mol) of binding energy superior to kojic acid, thus significantly breaking out of the standard’s threshold to highlight **4d**’s potential in tyrosinase inhibition.

**Figure 13:**
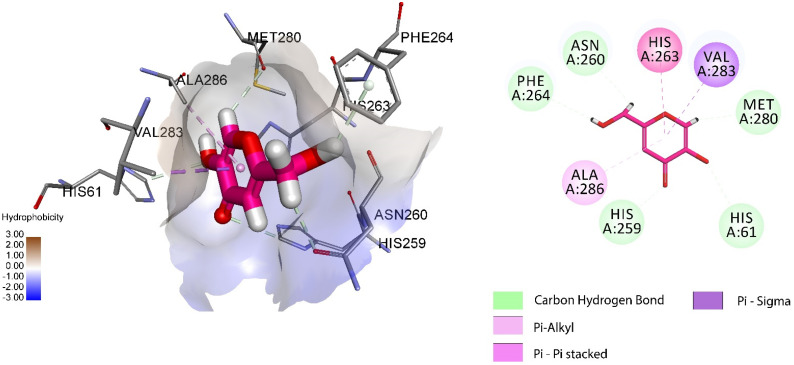
**Conformational analysis of kojic acid within the binding pocket:** Spatial configuration of binding mode of ligands in 3D space of binding pocket and their interaction profiles in 2D.

**Figure 14:**
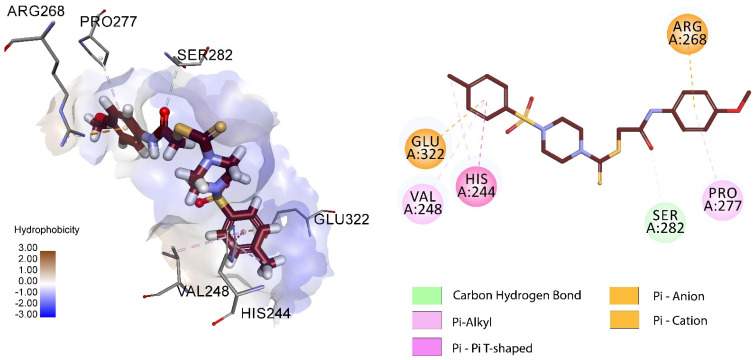
**Conformational analysis of 4d within the binding pocket:** Spatial configuration of binding mode of ligands in 3D space of binding pocket and their interaction profiles in 2D.

The 3D conformational analysis revealed that **4d** and kojic acid were stabilized by a diverse range of interactions with vital residues within the binding pocket. These ligands were found to arrange themselves in the binuclear copper ion vicinity to disrupt their interactions with the staggered lining of the histidine in the catalytic site, which may be responsible for their tyrosinase inhibitory potential. It was found that kojic acid stabilized its conformation by H-bonding with PHE264, ASN260, HIS259, HIS61, MET280 and had hydrophobic interactions with HIS263, VAL283, and ALA286 (Figure 13). Although **4d** disrupted the binuclear copper site, it shared distinct interaction profiles potentially by the high electron density of the catalytic site (Figure 14). It was revealed that **4d** was stabilized in the catalytic site by H-bonding with SER282 and by its hydrophobic interactions with HIS244, GLU322, VAL248, PRO277, and ARG268, thus highlighting its potential mechanism of action to inhibit tyrosinase catalytic activity.

## 4. Conclusions

In the current study, a series of ten novel 1-tosyl piperazine-dithiocarbamate acetamide derivatives **4a**–**j** was achieved in good to excellent yields (75–90%) and was evaluated for its in vitro tyrosinase inhibitory efficacy and hemolysis and thrombolysis activities. The 4-methoxy-containing piperazine-dithiocarbamate acetamide derivative **4d** displayed an excellent tyrosinase inhibitory activity (IC_50_ = 6.88 ± 0.11 µM) compared to the reference standard drugs kojic acid (30.34 ± 0.75 µM) and ascorbic acid (11.5 ± 1.00 µM), respectively. The tyrosinase activity in vitro results are in agreement with the in silico findings. These derivatives were also studied for their cytotoxic potential via hemolysis and thrombolysis. Among the target molecules, compound **4b** was found to be the least toxic (0.29 ± 0.01), while compound **4e** was recognized as a better thrombolytic agent (67.3 ± 0.2).

## Figures and Tables

**Scheme 1:**
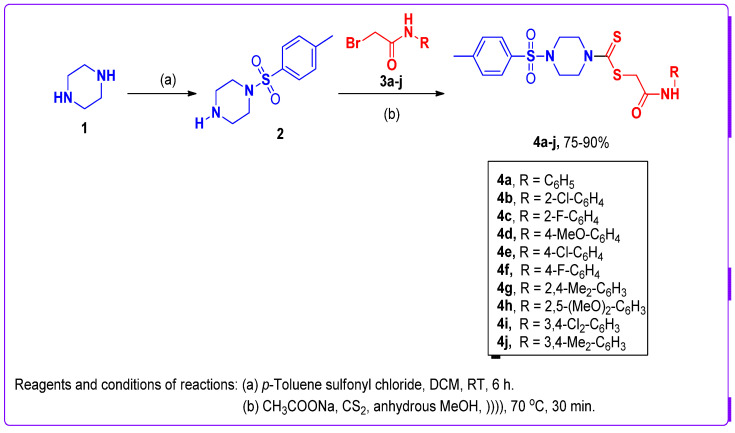
General outline for the synthesis of 1-tosyl piperazine-dithiocarbamate acetamide hybrids **4a**–**j**.

**Table 1:** Tyrosinase inhibition and hemolytic and thrombolytic assay of 1-tosyl piperazine-dithiocarbamate acetamide hybrids **4a**–**j**.

Compounds	–R	IC_50_ (µM) Tyrosinase Inhibition	% Hemolysis	% Thrombolysis
**4a**	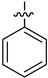	34.8 ± 0.99	0.55 ± 0.03	62.3 ± 0.41
**4b**	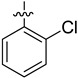	8.01 ± 0.11	0.29 ± 0.01	54.43 ± 0.23
**4c**	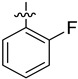	8.1 ± 0.30	6.6 ± 0.37	56.76 ± 0.15
**4d**	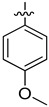	6.88 ± 0.11	15.6 ± 0.5	52.4 ± 0.20
**4e**	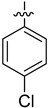	11.1 ± 0.4	7.5 ± 0.4	67.3 ± 0.2
**4f**	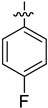	10.3 ± 0.26	4.53 ± 0.35	56.53 ± 0.55
**4g**	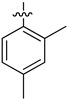	7.24 ± 0.15	1.53 ± 0.35	55.26 ± 0.15
**4h**	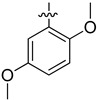	13.25 ± 0.11	11.32 ± 0.22	60.83 ± 0.14
**4i**	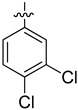	19.21 ± 0.47	12.8 ± 0.35	54.38 ± 0.53
**4j**	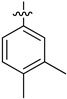	11.11 ± 0.10	2.56 ± 0.30	56.6 ± 0.23
**Kojic Acid**	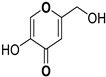	30.34 ± 0.75	--	--
**Ascorbic Acid** [4]	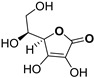	11.5 ± 1.00		
**DMSO**		--	0.0	0.57
**ABTS**		--	95.9	80

**Table 2:** Molecular docking analysis of compound **4d** and kojic acid with binding scores and interactions.

Compounds	Binding Score (S) Kcal/mol	Interacting Residues	Interaction Type
**Kojic acid**	−4.84	MET280, PHE264, ALA283, HIS263, ASN260, VAL283, HIS259	H-bonding, π- π stacked, π-alkyl, π-σ
**4d**	−7.3	GLU322, VAL248, HIS244, SER282, PRO277, ARG268	H-bonding, π-π T-Shaped, π-alkyl, π-cation, π-anion

## Data Availability

All the data obtained or analyzed during this study are reported in the article.

## Supplementary Materials

The following supporting information can be downloaded at: https://www.mdpi.com/article/10.3390/biomedicines11102739/s1, Figure S1: ^1^H NMR spectrum of compound **4a**; Figure S2: ^13^C NMR spectrum of compound **4a**; Figure S3: ^1^H NMR spectrum of compound **4b**; Figure S4: ^13^C NMR spectrum of compound **4b**; Figure S5: ^1^H NMR spectrum of compound **4c**; Figure S6: ^13^C NMR spectrum of compound **4c**; Figure S7: ^1^H NMR spectrum of compound **4d**; Figure S8: ^13^C NMR spectrum of compound **4d**; Figure S9: ^1^H NMR spectrum of compound **4e**; Figure S10: ^13^C NMR spectrum of compound **4e**; Figure S11: ^1^H NMR spectrum of compound **4f**; Figure S12: ^13^C NMR spectrum of compound **4f**; Figure S13: ^1^H NMR spectrum of compound **4g**; Figure S14: ^13^C NMR spectrum of compound **4g**; Figure S15: ^1^H NMR spectrum of compound **4h**; Figure S16: ^13^C NMR spectrum of compound **4h**; Figure S17: ^1^H NMR spectrum of compound **4i**; Figure S18: ^13^C NMR spectrum of compound **4i**; Figure S19: ^1^H NMR spectrum of compound **4j**; Figure S20: ^13^C NMR spectrum of compound **4j**.
