# Supplementary material for: Bacterial Tyrosinase Inhibition, Hemolytic and Thrombolytic Screening, and In Silico Modeling of Rationally Designed Tosyl Piperazine-Engrafted Dithiocarbamate Derivatives
Source: Biomedicines. 2023 Oct 10;11(10):2739. doi: 10.3390/biomedicines11102739 (PMC10603954; doi:10.3390/biomedicines11102739)
Supplement: Supplementary file 1 [file biomedicines-11-02739-s001.zip › biomedicines-2559420-supplementary.pdf]

Supporting information for

Bacterial Tyrosinase Inhibition, Hemolytic and Thrombolytic Screening and In Silico Modeling of Rationally Designed Tosyl Piperazine Engrafted Dithiocarbamate Derivatives

Ameer Fawad Zahoor [1], Freeha Hafeez [1,2], Asim Mansha [1,*], Shagufta Kamal [3], Muhammad Naveed Anjum [4], Zohaib Raza [5], Samreen Gul Khan [1], Jamila Javid [6], Ali Irfan [1] and Mashooq Ahmad Bhat [7,*]

1 Department of Chemistry, Government College University Faisalabad, 38000-Faisalabad, Pakistan. freeha.hafeez@riphahfsd.edu.pk (F.H.); asimmansha@gcuf.edu.pk (A.M.); fawad.zahoor@gcuf.edu.pk (A.F.Z.); samreengul@gcuf.edu.pk (S.G.K.); raialiirfan@gmail.com (A.I)
2 Department of Chemistry, Riphah International University Faisalabad, 38000-Faisalabad, Pakistan.
3 Department of Biochemistry, Government College University Faisalabad, 38000-Faisalabad, Pakistan. shaguftakamal81@gmail.com (S.K.)
4 Department of Applied Chemistry, Government College University Faisalabad, 38000-Faisalabad, Pakistan. anjumccj@hotmail.com (M.N.A.)
5 Department of Chemistry, School of Physical Sciences, University of Adelaide, Adelaide, South Australia 5000, Australia. zohaib.raza@adelaide.edu.au (Z.R)
6 Department of Chemistry, University of Sialkot, Sialkot, Pakistan. jamila.javaid@uskt.edu.pk (J.J.)
7 Department of Pharmaceutical Chemistry, College of Pharmacy, King Saud University, Riyadh 11451, Saudi Arabia. mabhat@ksu.edu.sa; (M.A.B.)
* Correspondence: asimmansha@gcuf.edu.pk (A.M.); mabhat@ksu.edu.sa;(M.A.B.)

^{1}H and ^{13}C NMR spectra of compound **4a-j**

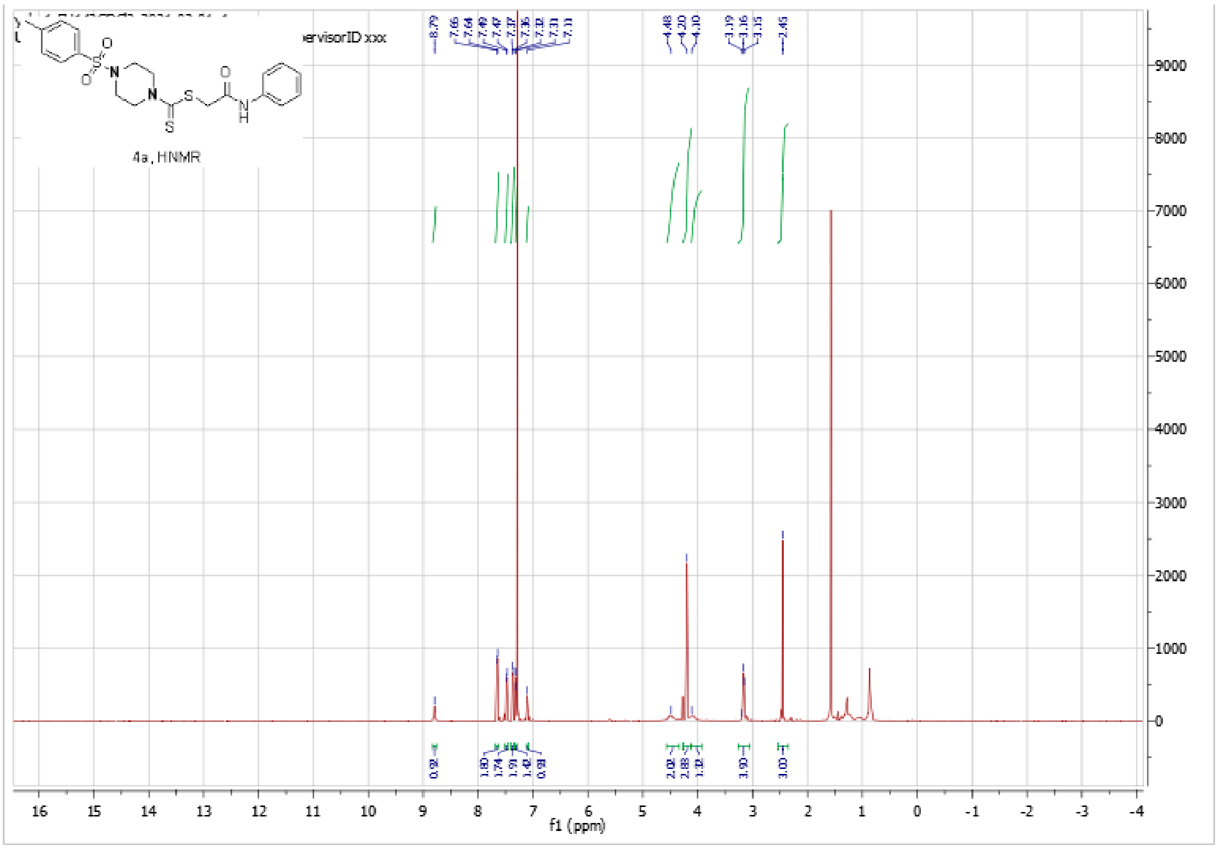

Figure S1: ^{1}H NMR spectrum of compound **4a**.

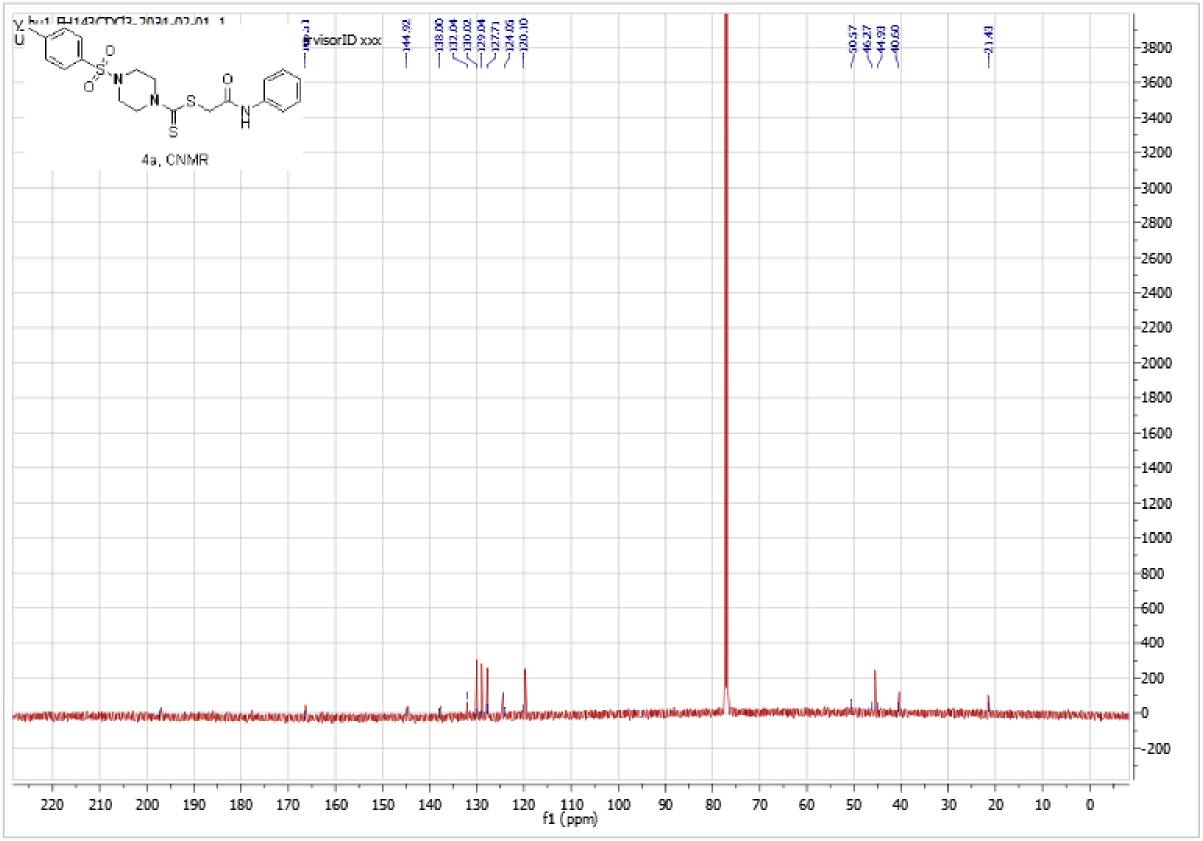

Figure S2: ^{13}C NMR spectrum of compound **4a**.

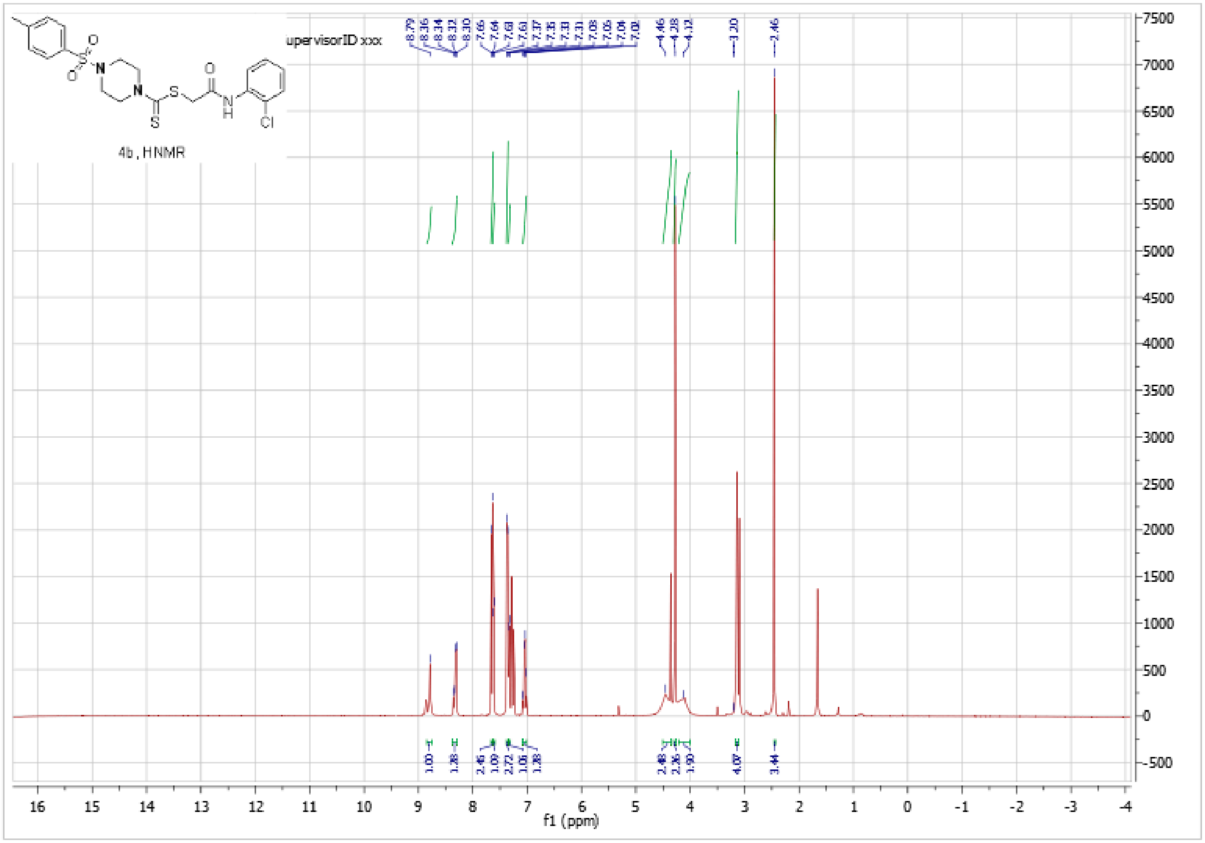

Figure S3: ^{1}H NMR spectrum of compound **4b**.

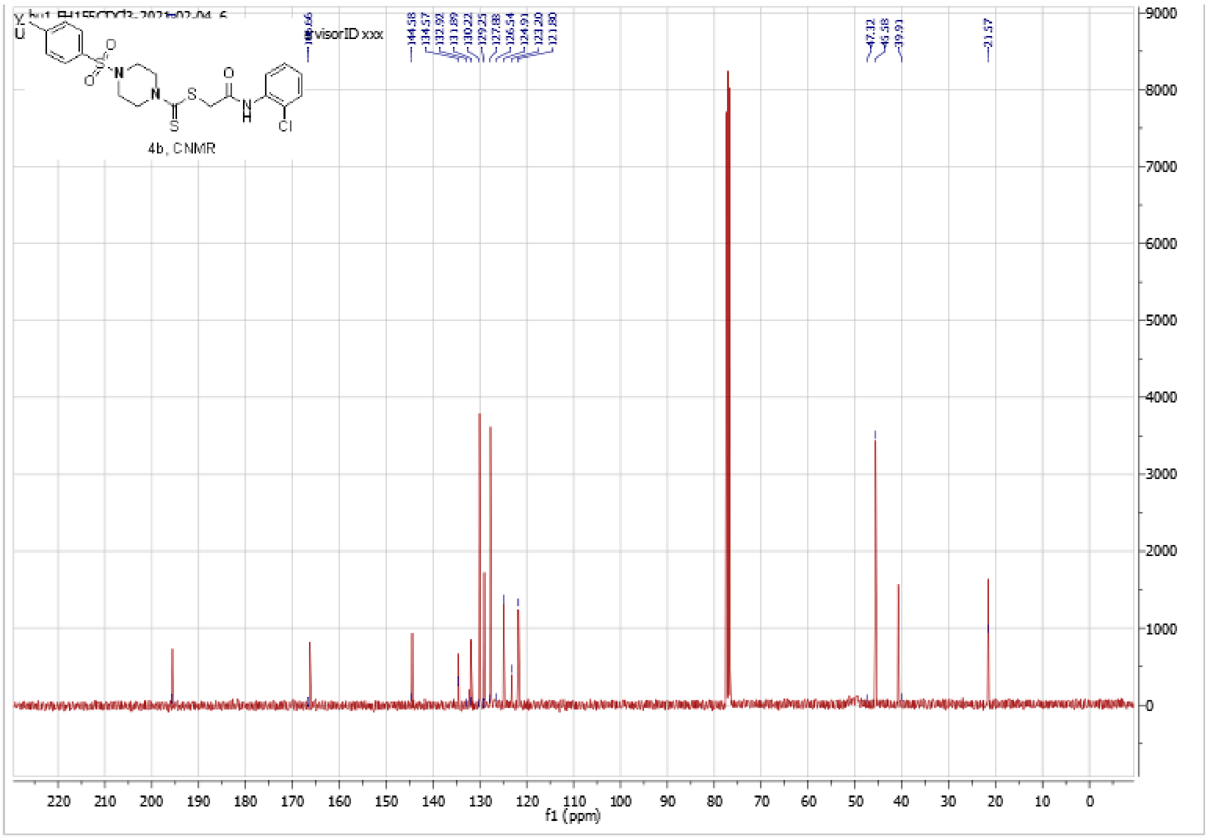

Figure S4: ^{13}C NMR spectrum of compound **4b**.

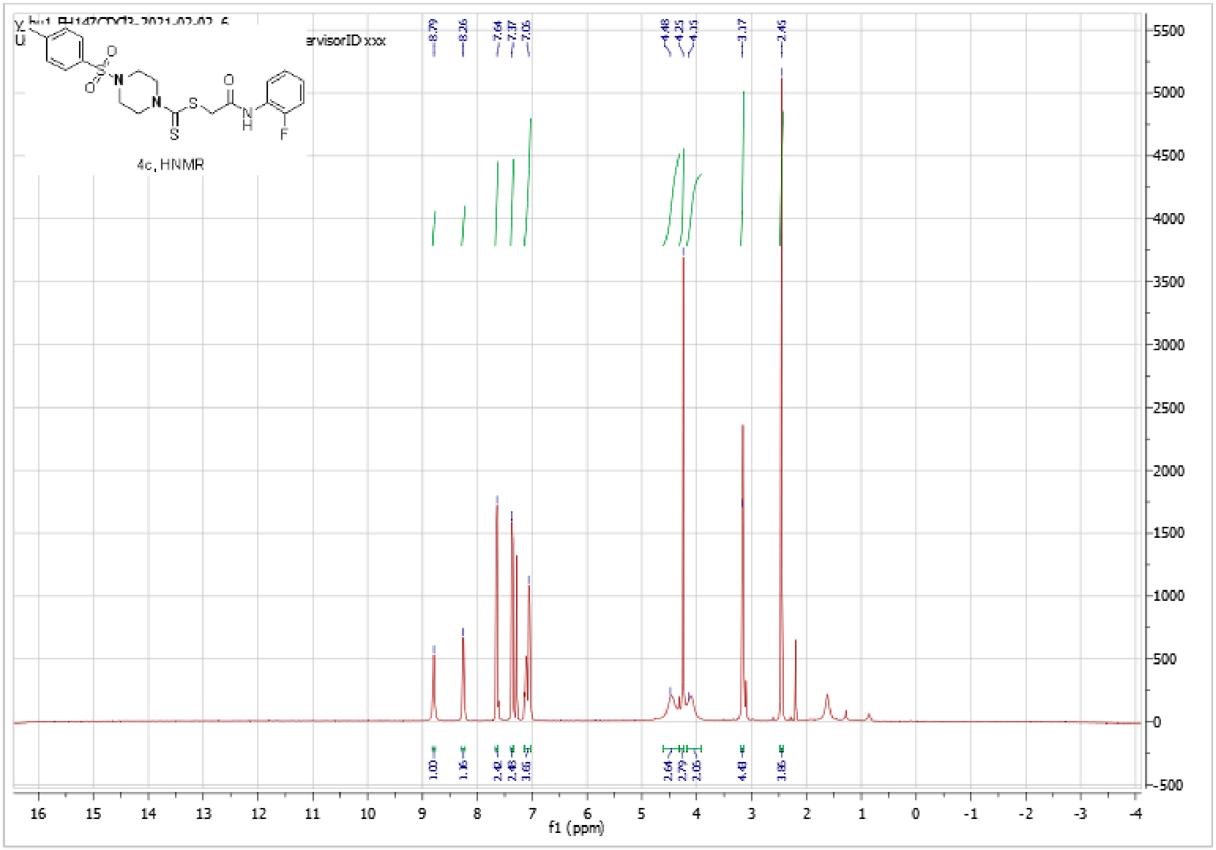

Figure S5: ^{1}H NMR spectrum of compound **4c**.

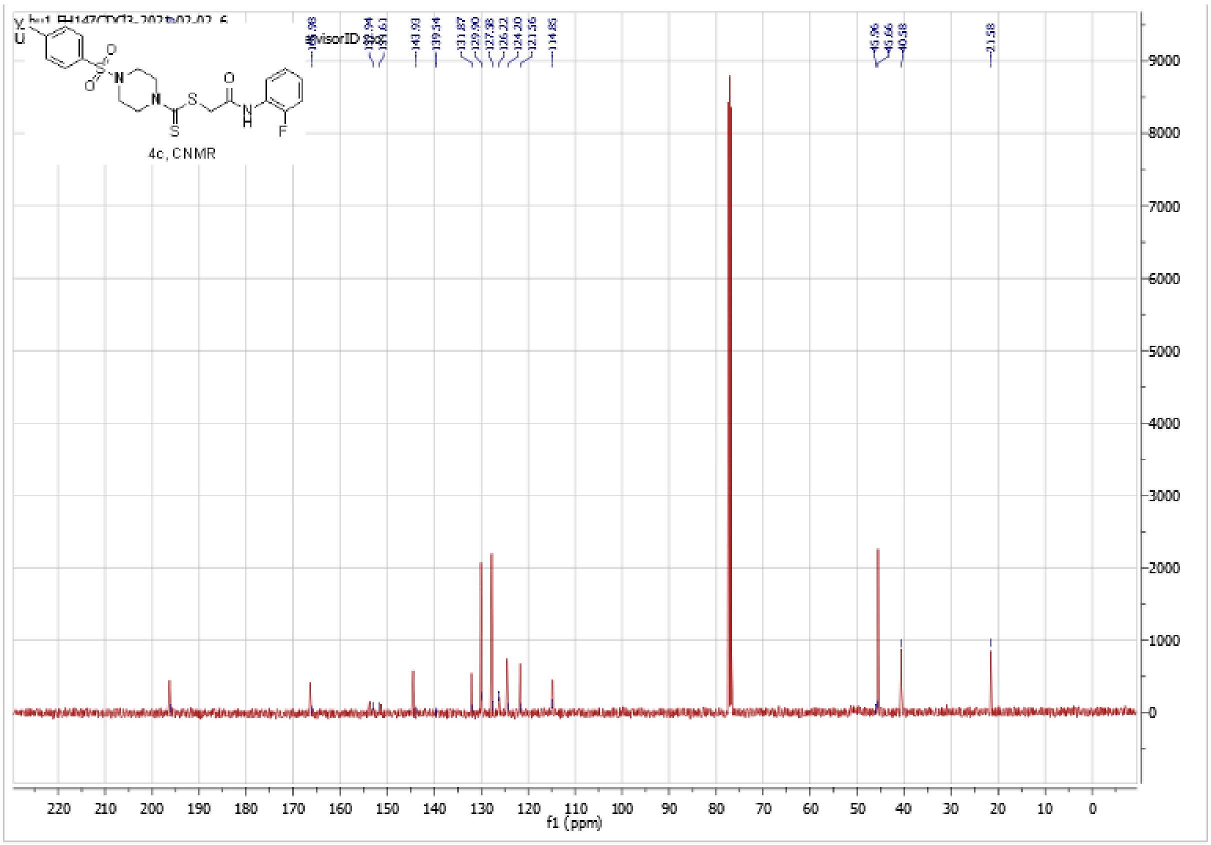

Figure S6: ^{13}C NMR spectrum of compound **4c**.

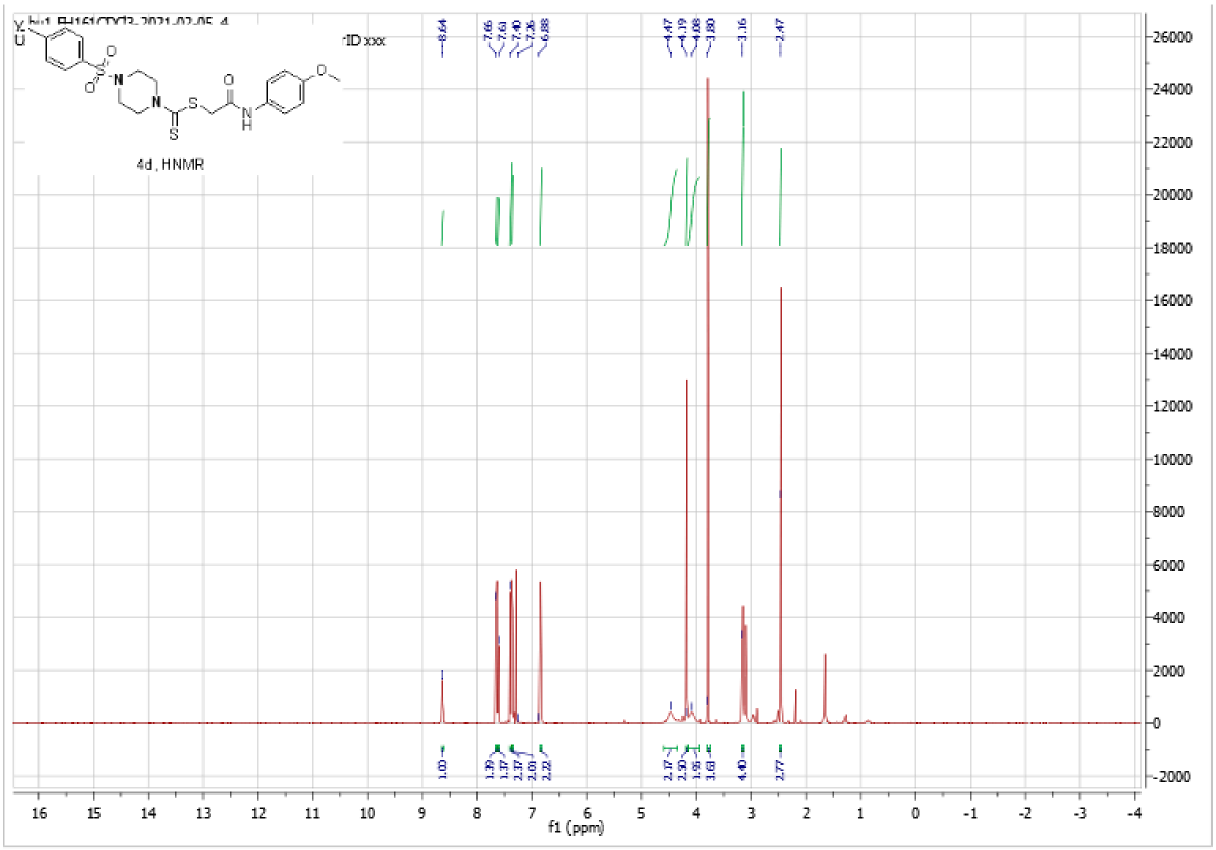

Figure S7: ^{1}H NMR spectrum of compound **4d**.

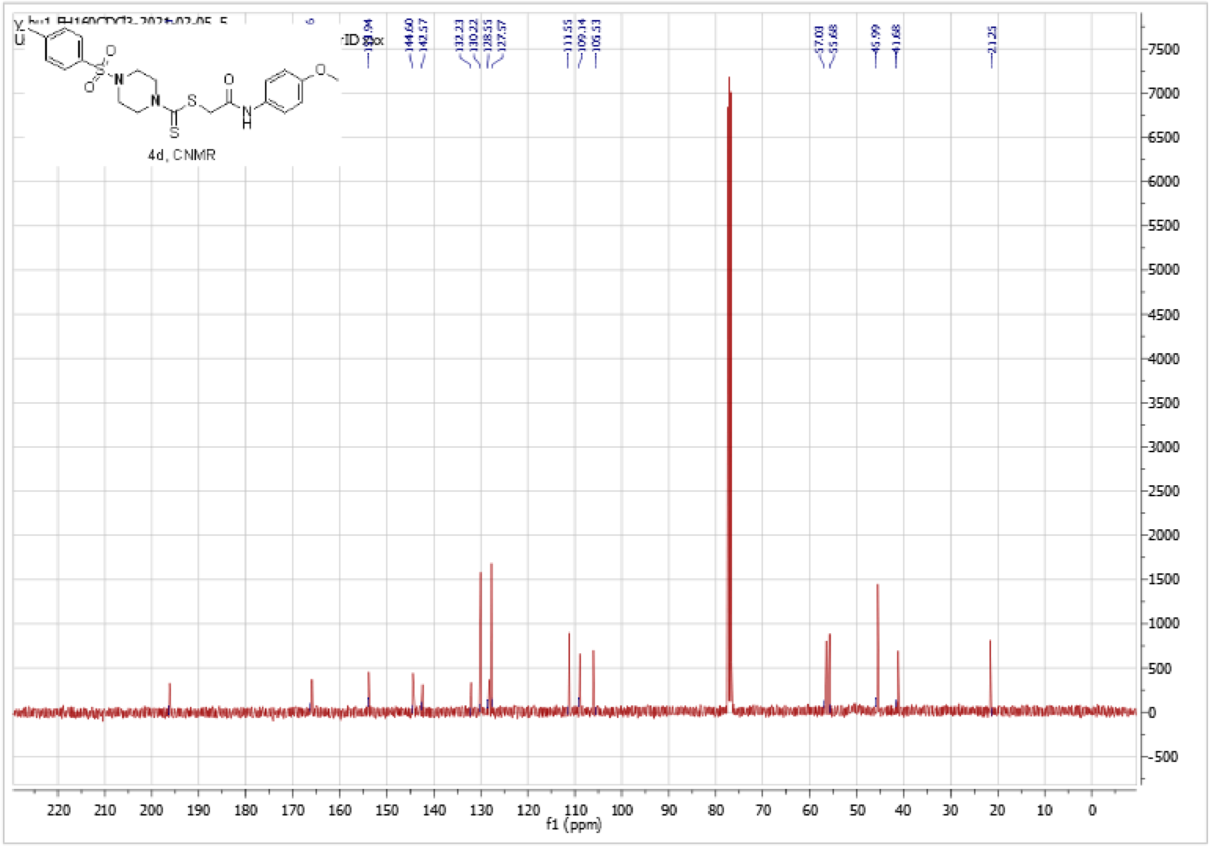

Figure S8: ^{13}C NMR spectrum of compound **4d**.

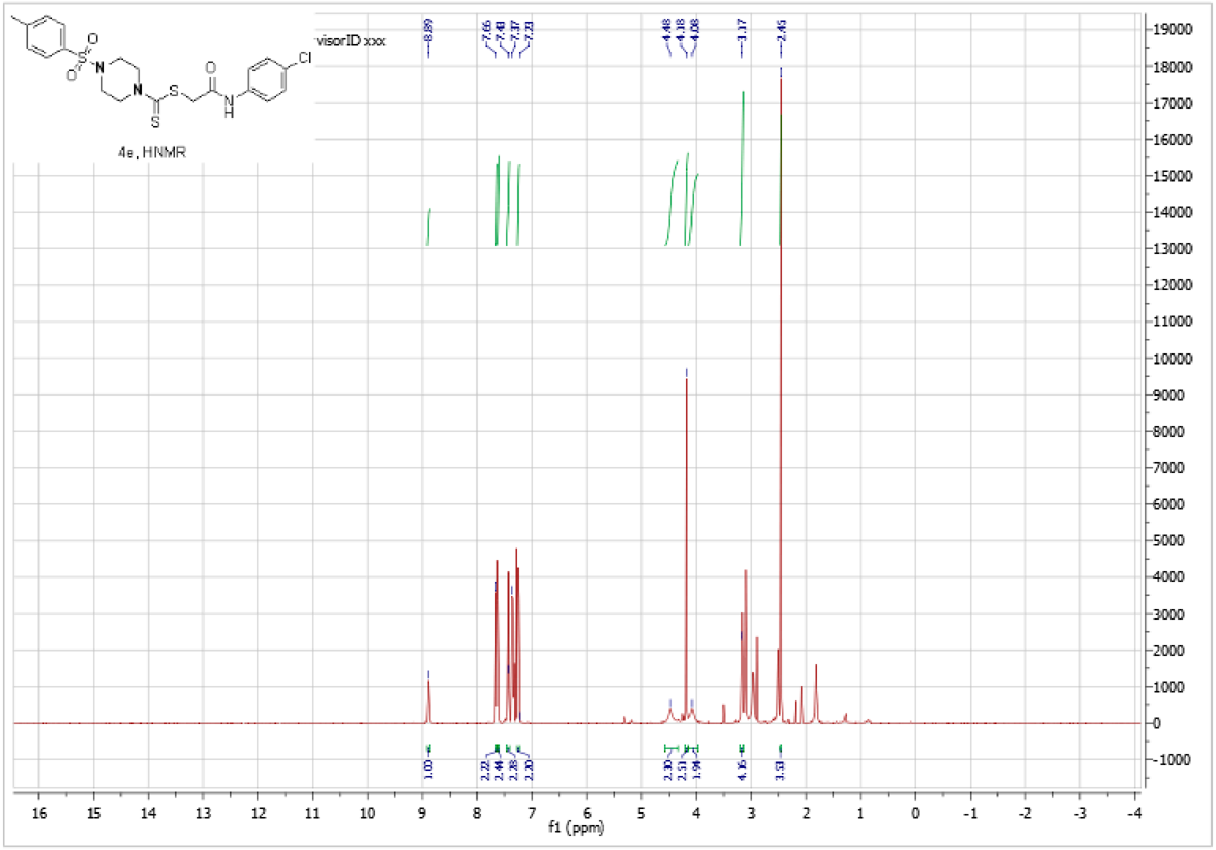

Figure S9: ^{1}H NMR spectrum of compound **4e**.

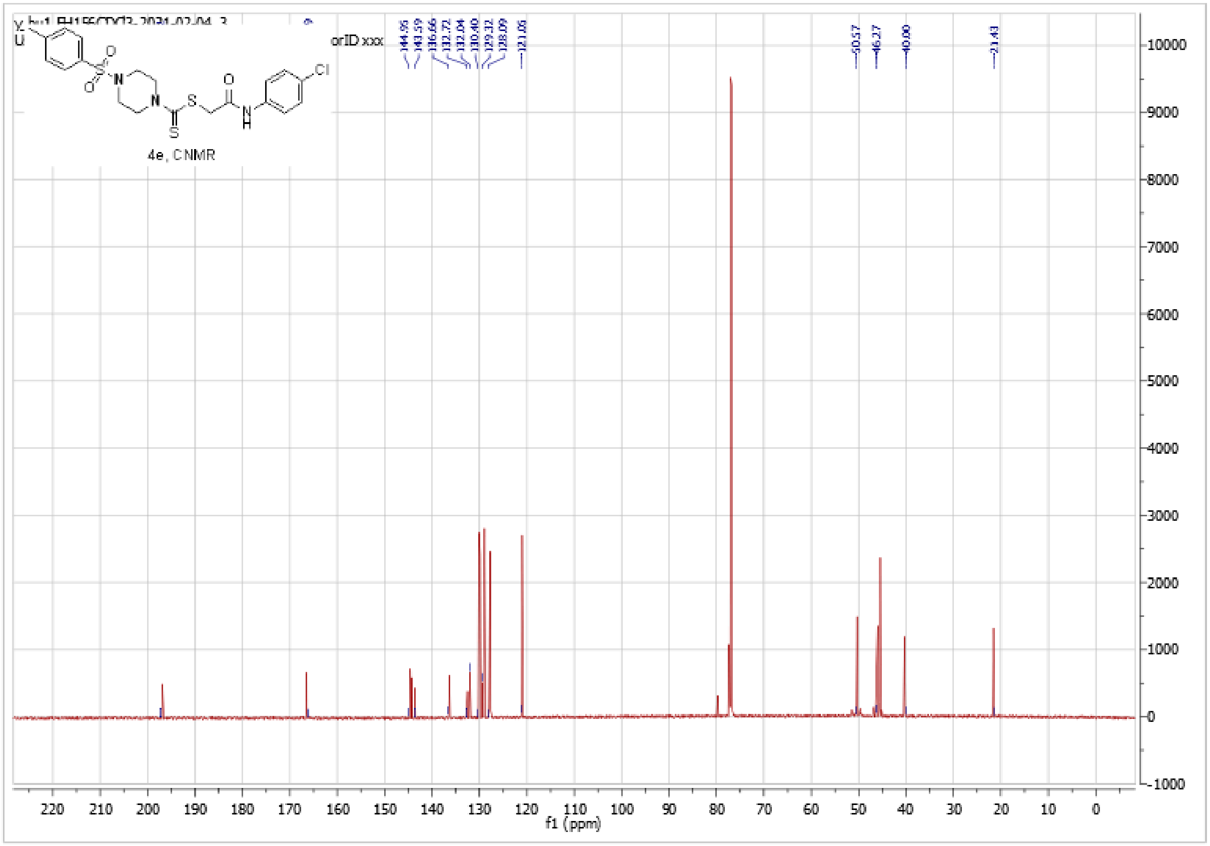

Figure S10: ^{13}C NMR spectrum of compound **4e**.

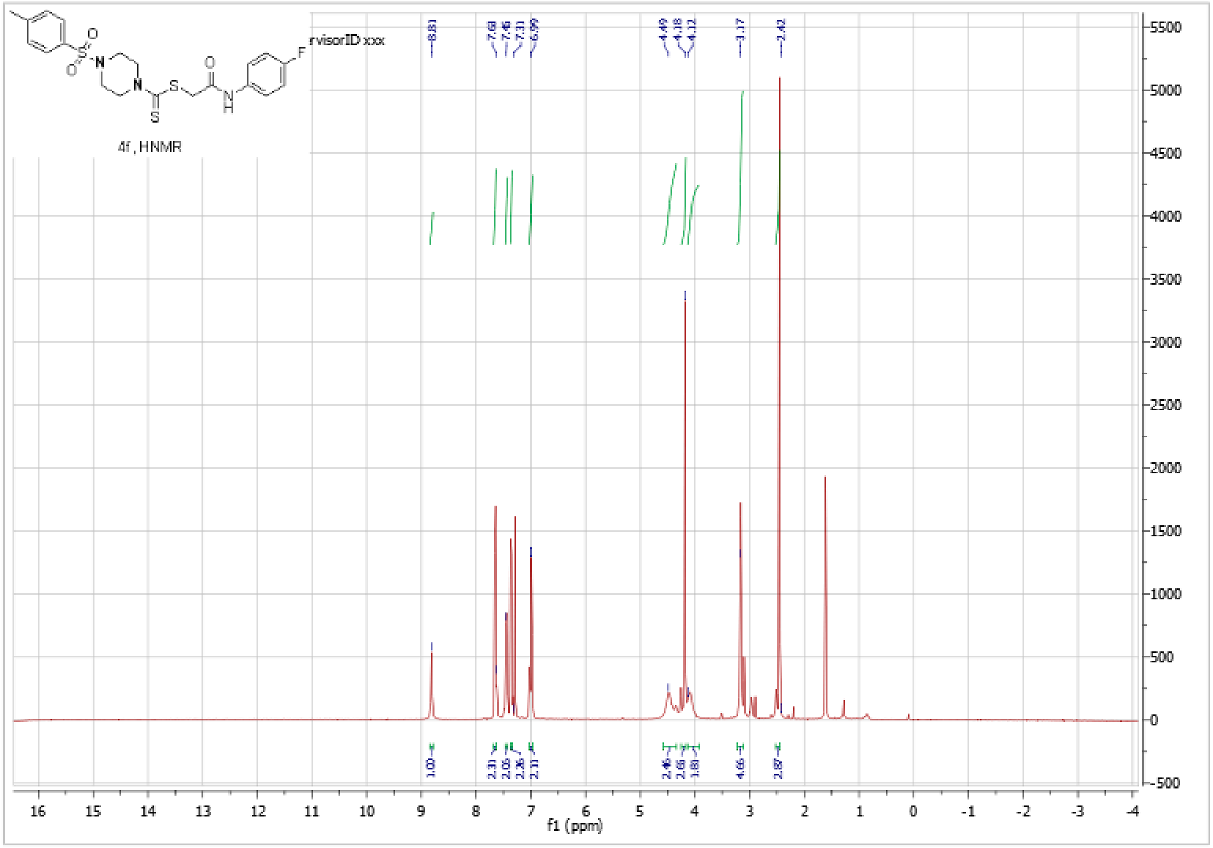

Figure S11: ^{1}H NMR spectrum of compound **4f**.

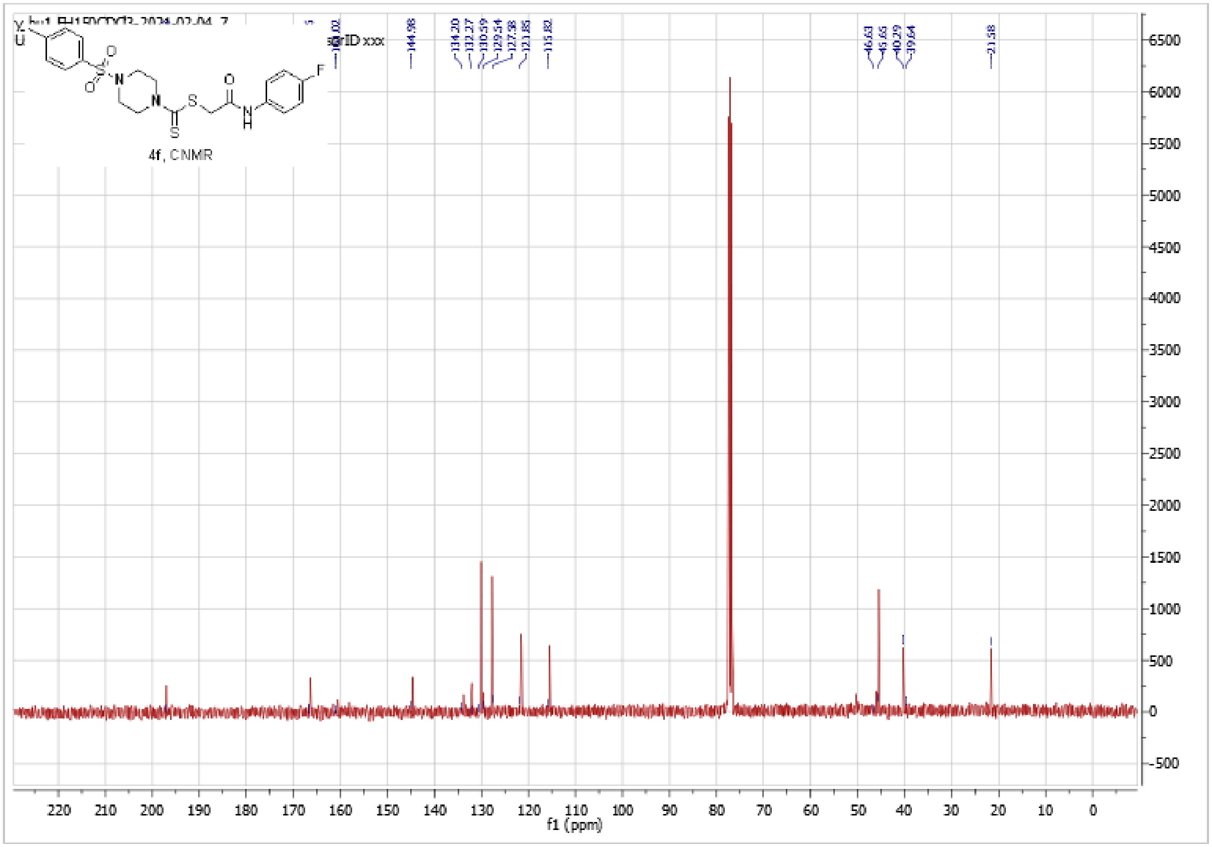

Figure S12: ^{13}C NMR spectrum of compound **4f**.

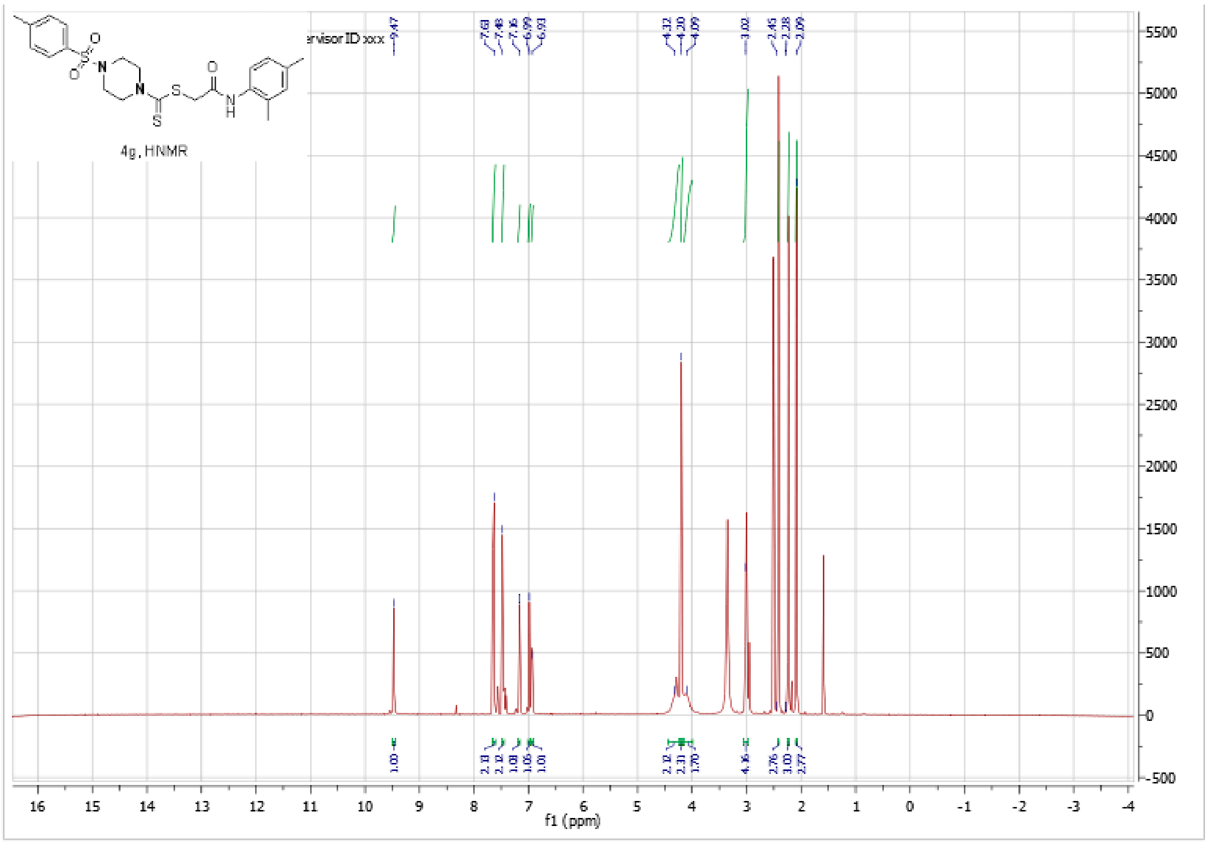

Figure S13: ^{1}H NMR spectrum of compound **4g**.

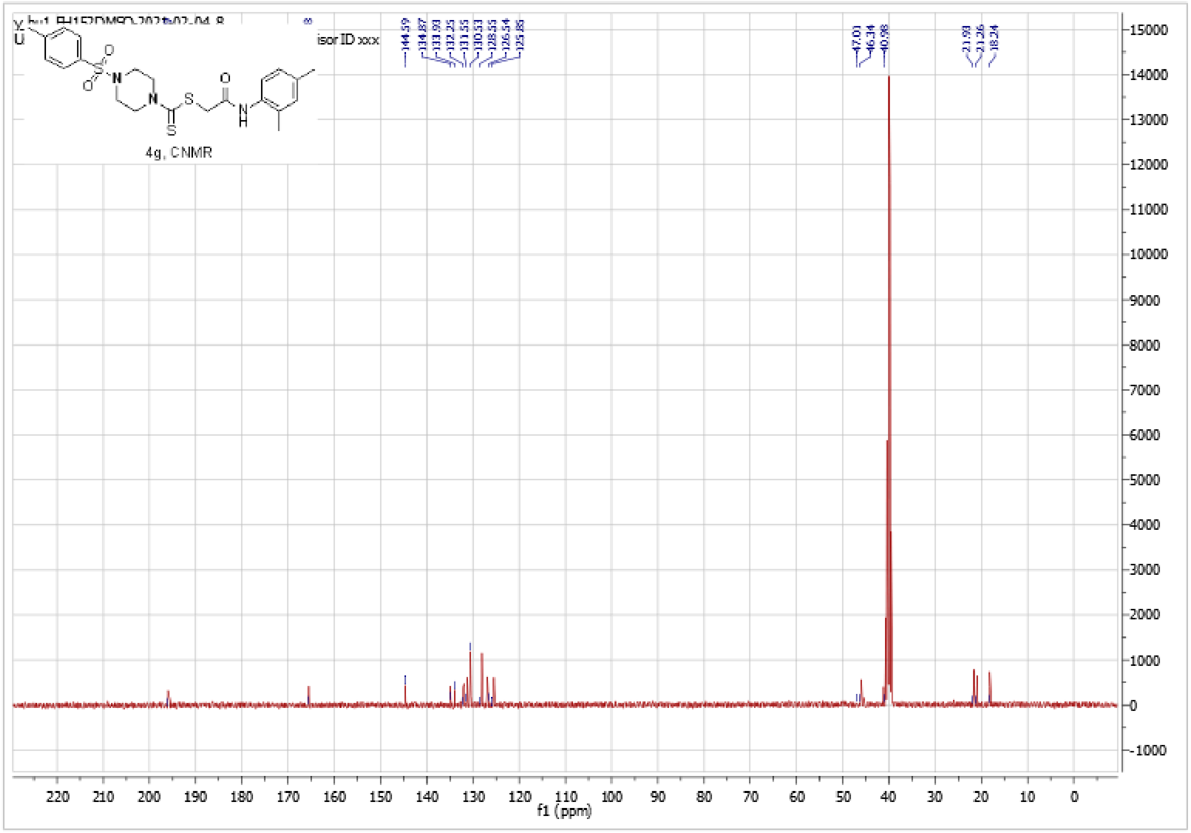

Figure S14: ^{13}C NMR spectrum of compound **4g**.

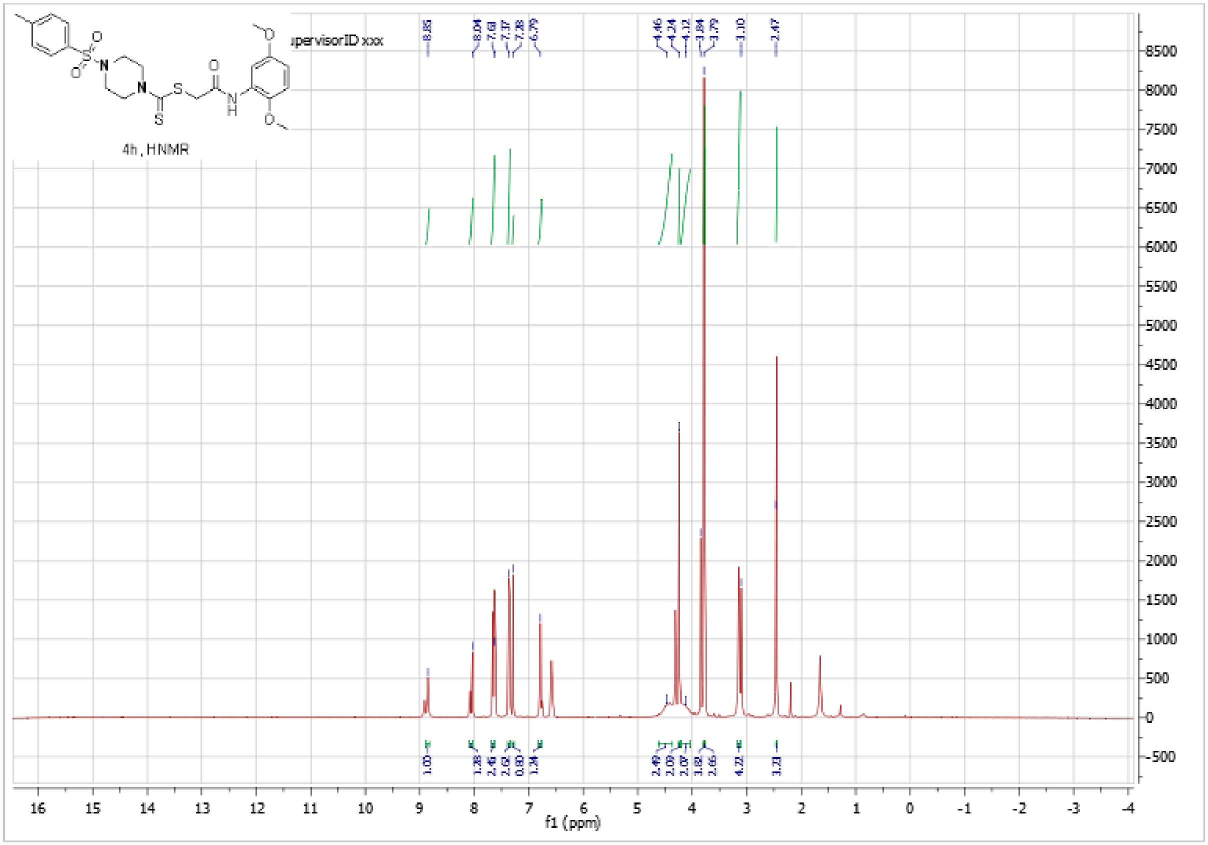

Figure S15: ^{1}H NMR spectrum of compound **4h**.

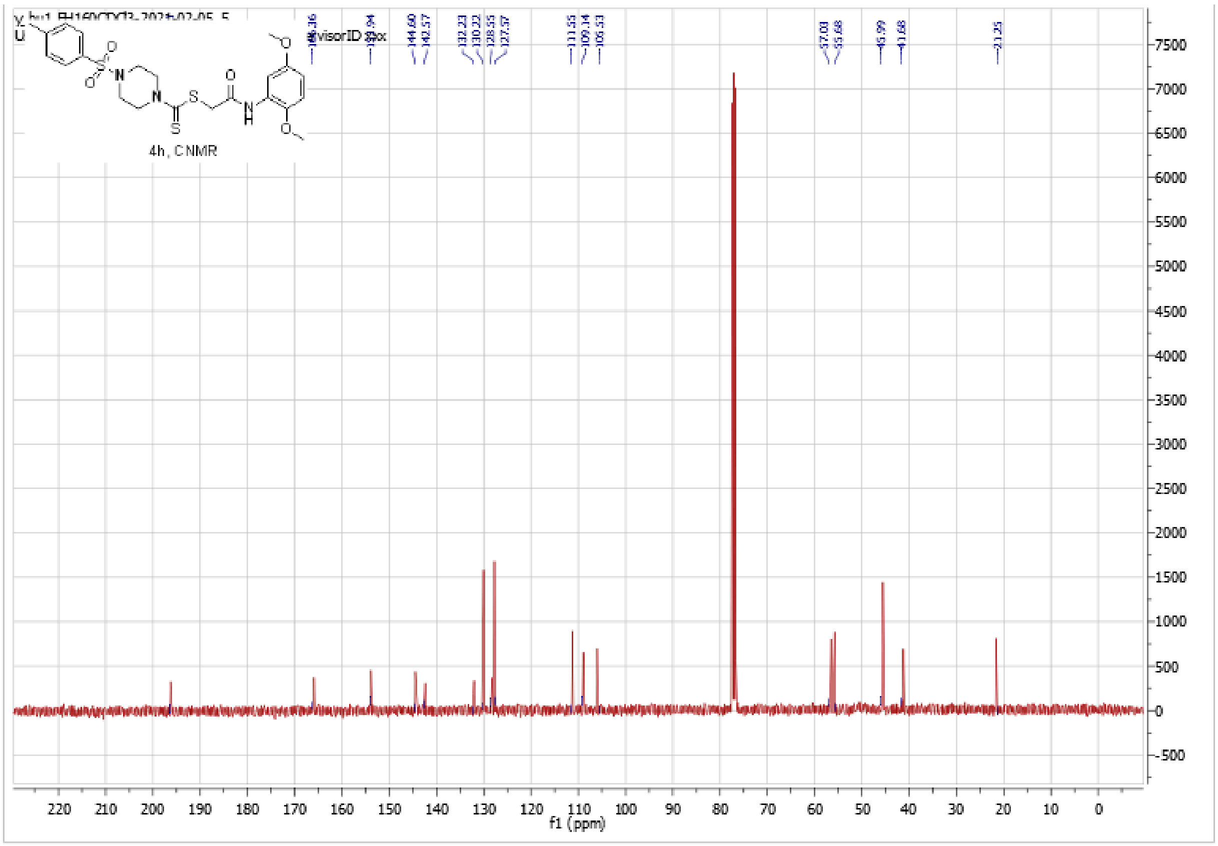

Figure S16: ^{13}C NMR spectrum of compound **4h**.

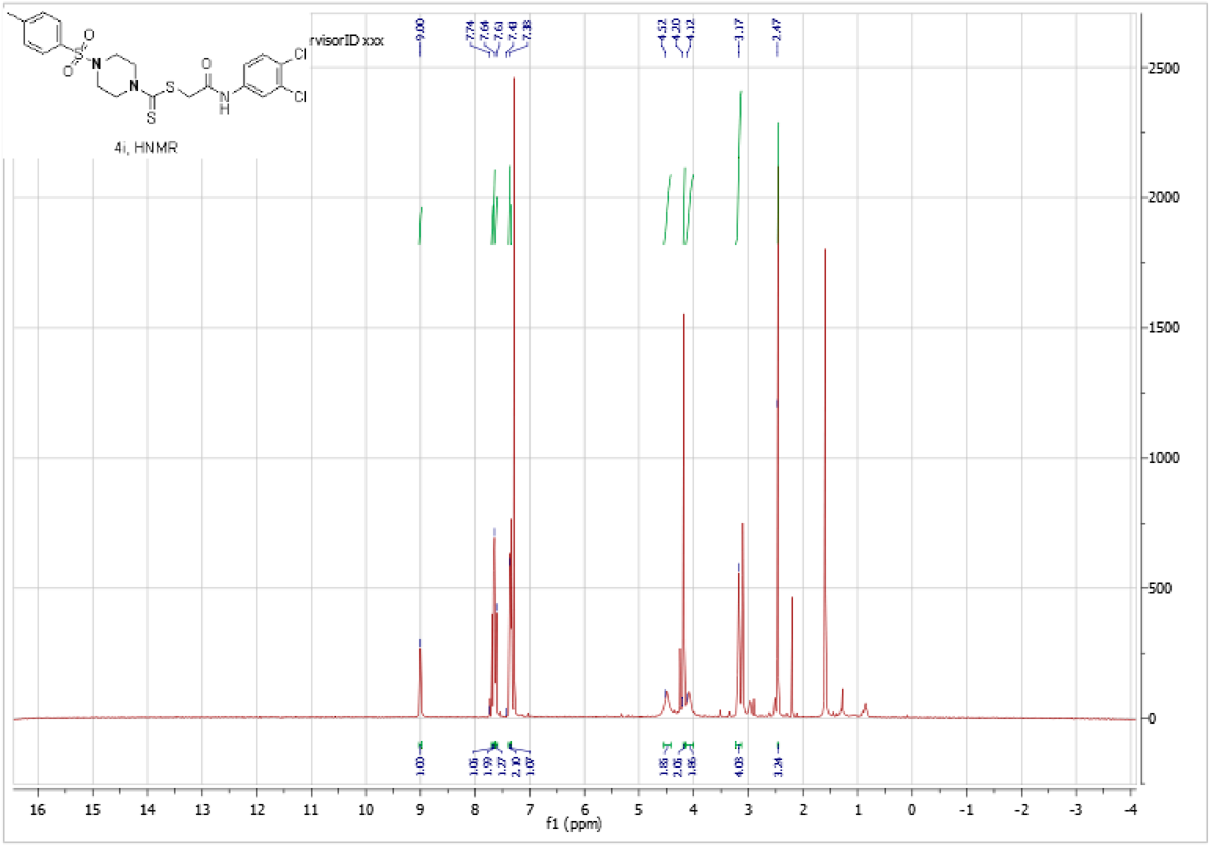

Figure S17: ^{1}H NMR spectrum of compound **4i**.

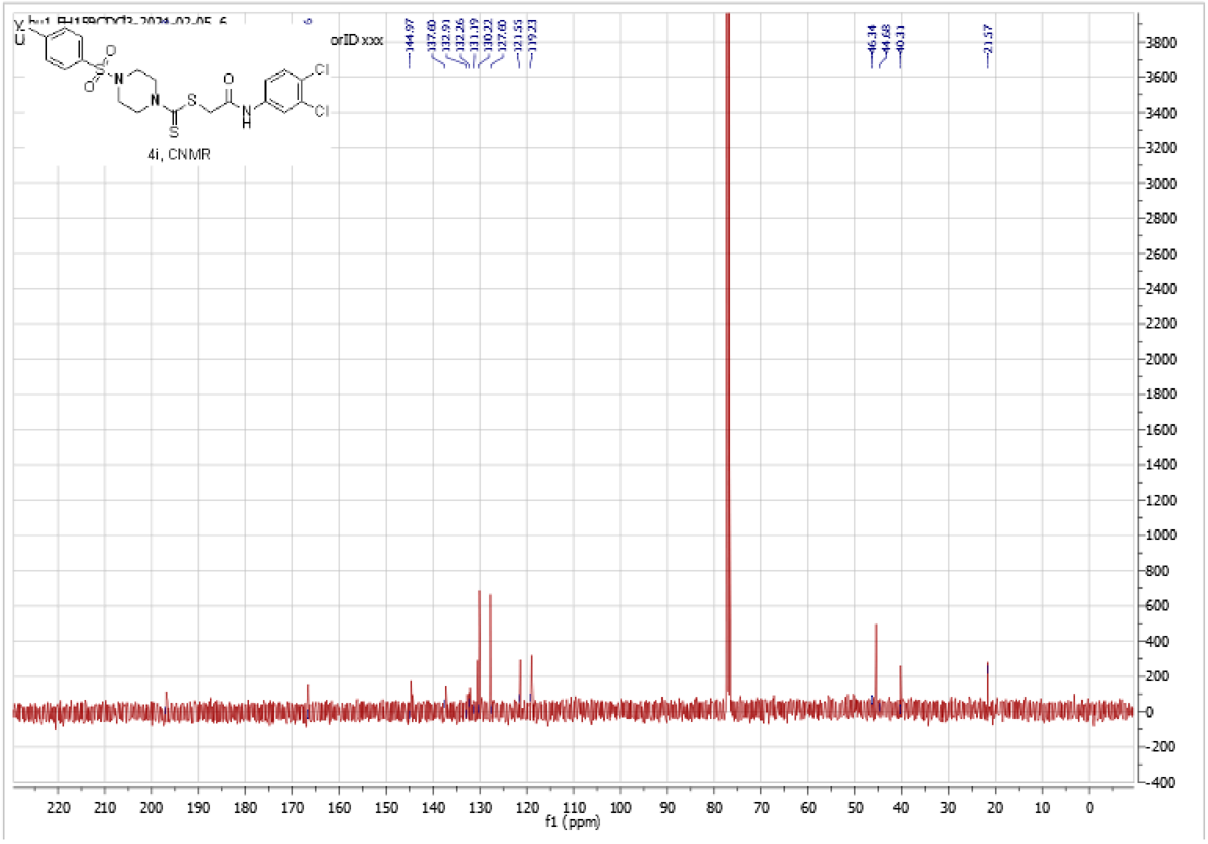

Figure S18: ^{13}C NMR spectrum of compound **4i**.

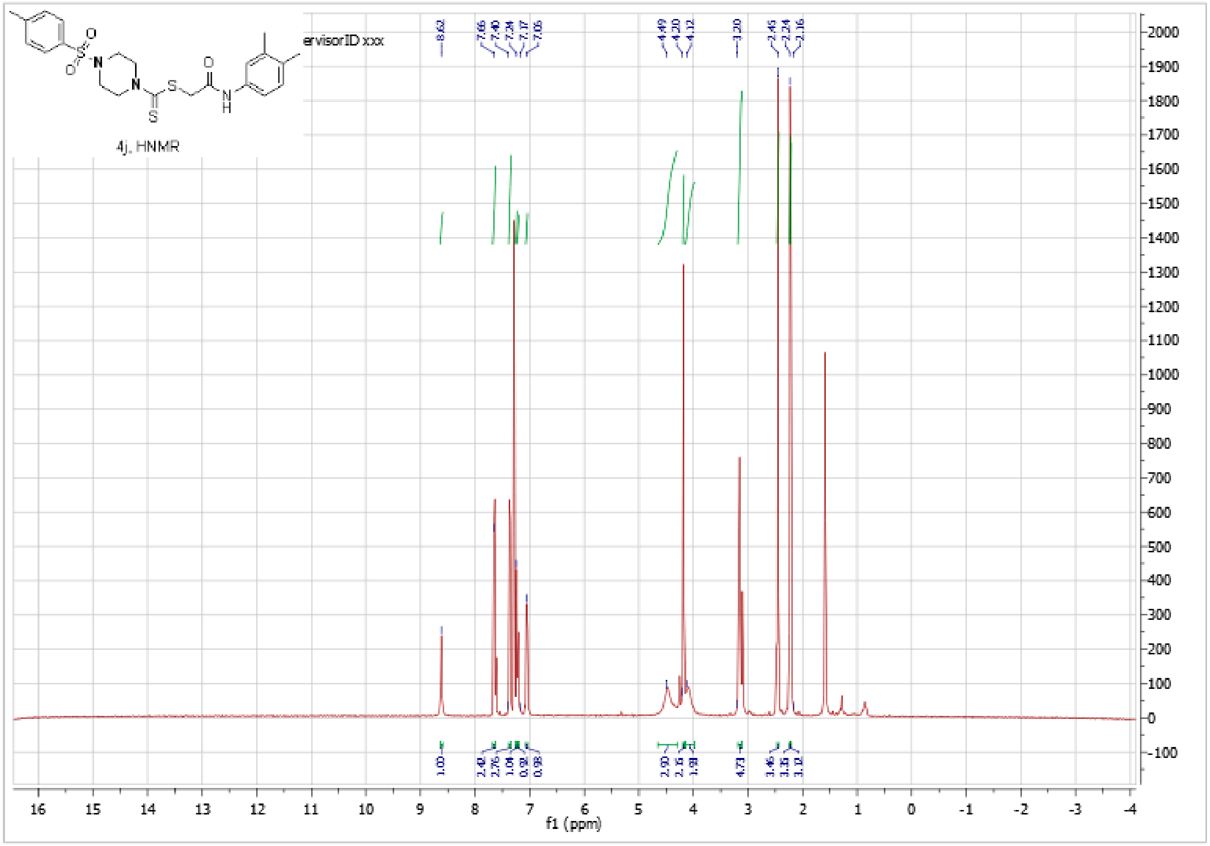

Figure S19: ^{1}H NMR spectrum of compound **4j**.

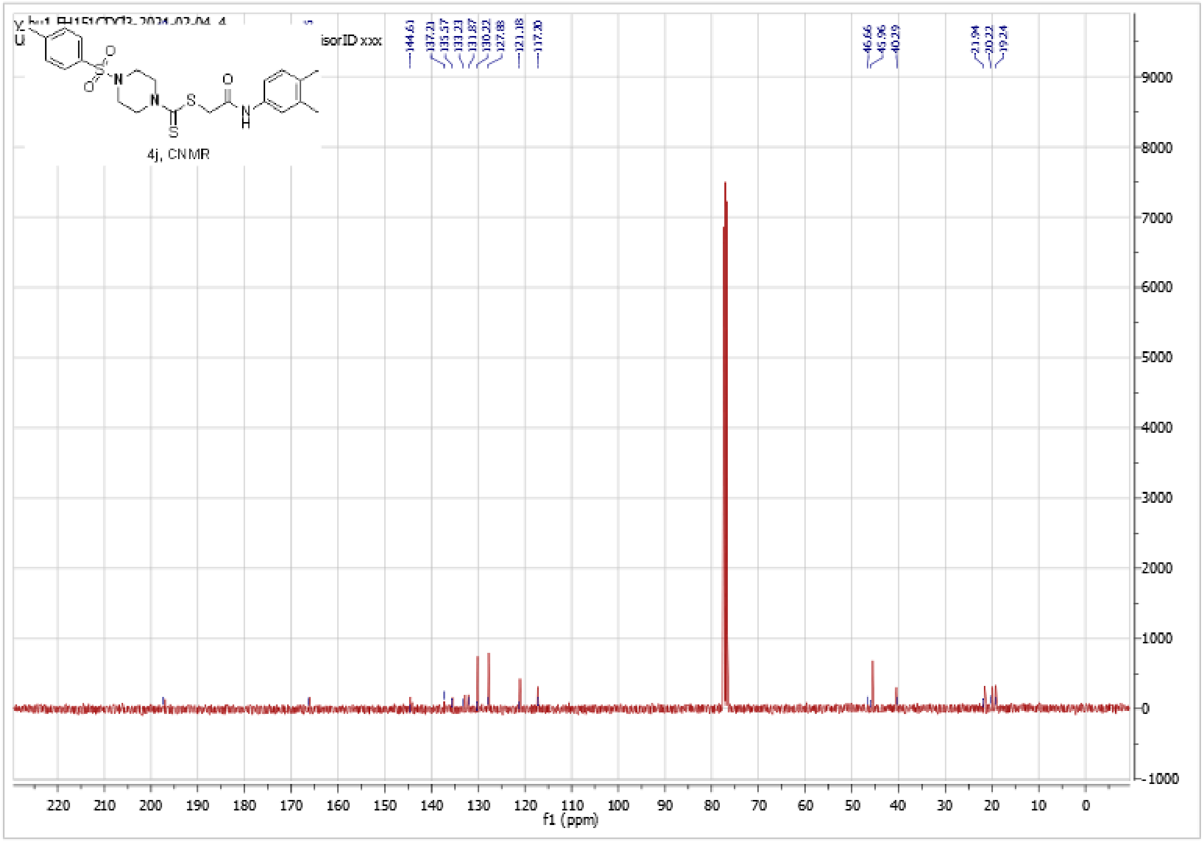

Figure S20: ^{13}C NMR spectrum of compound **4j**.
